# Small-scale spatiotemporal epidemiology of notifiable infectious diseases in China: a systematic review

**DOI:** 10.1186/s12879-022-07669-9

**Published:** 2022-09-05

**Authors:** Junyao Zheng, Guoquan Shen, Siqi Hu, Xinxin Han, Siyu Zhu, Jinlin Liu, Rongxin He, Ning Zhang, Chih-Wei Hsieh, Hao Xue, Bo Zhang, Yue Shen, Ying Mao, Bin Zhu

**Affiliations:** 1 China Institute for Urban Governance, Shanghai Jiao Tong University, Shanghai, China; 2 School of Public Administration and Policy, Renmin University of China, Beijing, China; 3 School of Public Policy and Administration, Xi’an Jiaotong University, Xi’an, China; 4 School of Public Health and Emergency Management, Southern University of Science and Technology, Shenzhen, 518055 Guangdong China; 5 School of Public Policy and Administration, Northwestern Polytechnical University, Xi’an, China; 6 Vanke School of Public Health, Tsinghua University, Beijing, China; 7 MRC Centre for Global Infectious Disease Analysis and the Abdul Latif Jameel Institute for Disease and Emergency Analytics, School of Public Health, Imperial College, London, UK; 8 Department of Public Policy, City University of Hong Kong, Hong Kong, China; 9 Freeman Spogli Institute for International Studies, Stanford University, Stanford, CA USA; 10 Department of Earth System Science, Tsinghua University, Beijing, China; 11 Laboratory for Urban Future, School of Urban Planning and Design, Peking University Shenzhen Graduate School, Shenzhen, China; 12 School of International and Public Affairs, Shanghai Jiao Tong University, Shanghai, China

**Keywords:** Notifiable infectious diseases, Spatiotemporal epidemiology, Spatial epidemiology, Geographical scale, China

## Abstract

**Background:**

The prevalence of infectious diseases remains one of the major challenges faced by the Chinese health sector. Policymakers have a tremendous interest in investigating the spatiotemporal epidemiology of infectious diseases. We aimed to review the small-scale (city level, county level, or below) spatiotemporal epidemiology of notifiable infectious diseases in China through a systematic review, thus summarizing the evidence to facilitate more effective prevention and control of the diseases.

**Methods:**

We searched four English language databases (PubMed, EMBASE, Cochrane Library, and Web of Science) and three Chinese databases (CNKI, WanFang, and SinoMed), for studies published between January 1, 2004 (the year in which China’s Internet-based disease reporting system was established) and December 31, 2021. Eligible works were small-scale spatial or spatiotemporal studies focusing on at least one notifiable infectious disease, with the entire territory of mainland China as the study area. Two independent reviewers completed the review process based on the Preferred Reporting Items for Systematic Reviews and Meta-Analyses guidelines.

**Results:**

A total of 18,195 articles were identified, with 71 eligible for inclusion, focusing on 22 diseases. Thirty-one studies (43.66%) were analyzed using city-level data, 34 (47.89%) were analyzed using county-level data, and six (8.45%) used community or individual data. Approximately four-fifths (80.28%) of the studies visualized incidence using rate maps. Of these, 76.06% employed various spatial clustering methods to explore the spatial variations in the burden, with Moran’s I statistic being the most common. Of the studies, 40.85% explored risk factors, in which the geographically weighted regression model was the most commonly used method. Climate, socioeconomic factors, and population density were the three most considered factors.

**Conclusions:**

Small-scale spatiotemporal epidemiology has been applied in studies on notifiable infectious diseases in China, involving spatiotemporal distribution and risk factors. Health authorities should improve prevention strategies and clarify the direction of future work in the field of infectious disease research in China.

**Supplementary Information:**

The online version contains supplementary material available at 10.1186/s12879-022-07669-9.

## Background

In traditional epidemiology, patterns are identified by examining the characteristics of person, place, and time, whereas in modern epidemiology, spatial perspective is incorporated into research designs and models [1]. In the twenty-first century, the field of spatial epidemiology has evolved rapidly and is currently playing an important role in monitoring diseases, assessing the effectiveness of control and prevention measures, identifying high-risk regions, and helping authorities develop public health policies [2, 3].

China, the world's most populous nation, has greatly reduced its infectious disease burden over the past two decades. However, infectious diseases remain one of the major challenges faced by the Chinese health sector [4, 5]. The most recent coronavirus disease 2019 (COVID-19) pandemic has raised concerns about infectious diseases. To further monitor and respond to possible infectious disease outbreaks, China created a list of notifiable infectious diseases (Additional file 1: Table S1, two in Class A, 27 in Class B, and 11 in Class C), characterized by wide distribution, high prevalence, or severe threats, which is specified by the Law of the People’s Republic of China on the Prevention and Treatment of Infectious Diseases. The shift in infectious disease surveillance from paper forms to electronic documents also supports the use of data to promptly detect and respond to infectious disease outbreaks [6]. Before the mid-1980s, paper-based notifications were distributed monthly by post. Digitized monthly reports were adopted from the mid-1980s and through 2003. In 2004, a real-time Internet-based China Information System for Disease Control and Prevention (CISDCP) was established by the China CDC, which improved the timeliness and accuracy of surveillance [6]. The CISDCP collects patient case reports for all notifiable diseases from all medical institutions in China.

Spatial disparities exist in the distribution of infectious diseases owing to differences in economic development levels, population density, meteorological factors, and more [7, 8]. There is tremendous interest from policymakers, public health practitioners, and researchers in understanding the spatiotemporal epidemiology of notifiable infectious diseases in China. Many studies have focused on such diseases, including but not limited to sexually transmitted infections [9], intestinal infectious diseases [10], respiratory infectious diseases [10], and viral hepatitis (A, B, C, E, and more) [11]. However, these studies targeted different diseases, covering different periods and adopting different research scales (province, city, and county levels). Policymakers need systematic reviews to help select, appraise, and synthesize research findings. To our knowledge, there have been several systematic reviews on the spatiotemporal characteristics and analytic mechanism of specific infectious diseases globally, such as malaria [12], tuberculosis [13], leptospirosis [14], and dengue [15]. However, these reviews did not summarize and compare the transmission characteristics of infectious diseases and only included descriptive statistics because the spatial scale of the included studies was inconsistent [16]. Systematic reviews of the small-scale spatiotemporal epidemiology of infectious diseases in a country could give policymakers insights about how different infectious diseases share similarities and differences in the distribution of cases and determinants. In addition, exploring and summarizing the spatiotemporal dynamics of notifiable infectious diseases could provide local governments with important reference values for standardizing epidemic prevention protocols and equip policymakers with the information required to confidently determine the probable effects of area-targeted prevention and control policies.

This study aimed to systematically review the spatiotemporal epidemiology of notifiable infectious diseases in China. We focused on small-scale analyses, as considerable current interest in the field of spatial epidemiology can be observed in a smaller scale of research.

## Methods

### Data sources and search strategy

A broad search strategy using multiple electronic literature databases was employed to minimize the risk of bias. We searched seven literature databases from January 1, 2004 (the year the CISDCP was established) to December 31, 2021, including four English databases (Web of Science, PubMed, EMBASE, and Cochrane Library) and three Chinese databases (CNKI, WanFang, and SinoMed), following the Preferred Reporting Items for Systematic Reviews and Meta-Analyses (PRISMA) guidelines [17].

The search syntax (Additional file 1: Supplemental file 3) was based on a combination of the following terms with the Boolean phrase ‘OR’ within groups or ‘AND’ between groups: (1) location-related terms, such as China and Chinese; (2) spatial analysis related terms, such as spatial, spatiotemporal, and geographical; (3) infectious disease-related terms, such as infectious diseases, epidemic, and virus diseases; and (4) notifiable infectious diseases in China-related terms, such as plague, cholera, and SARS.

EndNote Software Version 20 was employed to manage the citations.

### Eligibility criteria

The following eligibility criteria were defined: (1) Chinese and English epidemiological articles published from January 1, 2004 to December 31, 2021; (2) the application of spatial analysis or mapping; (3) the research scope limited to Mainland China; (4) research scales at the city level, county level, or below; (5) data extracted from the Chinese Centre for Disease Control and Prevention, National Health Commission of the People’s Republic of China, or other authorities; (6) related to one or more notifiable infectious diseases in China; and (7) published in a peer-reviewed journal.

The exclusion criteria were as follows: (1) studies including other countries or regions outside China; (2) a research scale at the provincial level; (3) a research scope of only specific provinces; (4) disease that were not notifiable infectious diseases in China; (5) qualitative articles; (6) review articles; and (7) editorials or published letters.

### Study selection

After deduplication, two reviewers (JZ and GS) independently screened titles and abstracts. Disagreements were resolved by consulting a third reviewer (BZ) to make the decision. Subsequently, two reviewers (SH and NZ) examined the full text and assessed it according to the set criteria. Finally, all reviewers participated in the data extraction.

### Data collection process

Information about the spatial methods and outcomes was extracted from each included study (shown in Table 1). Descriptive details obtained included first author, publication year, type of infectious disease, period of analysis, research scale, study type, spatial methods used, study aspects, and risk factors.

**Table 1:** Contextual details of studies included

References	Infectious disease	Period of analysis	Research scale	Study type	Study aspects	Risk factors
[18]	Hemorrhagic fever	1994–1998	County level	Longitudinal	Characteristics	N/A
[19]	HFMD	2008.1–2009.10	City level	Longitudinal	Characteristics	N/A
[20]	HFMD	2008.5	County level	Cross-sectional	Characteristics; Risk factors	Population density and climate
[21]	HFMD	2008.5–2011.12	County level	Longitudinal	Characteristics	N/A
[22]	Syphilis	2004–2010	County level	Longitudinal	Characteristics	N/A
[23]	Rabies	2005–2011	Individual level	Longitudinal	Characteristics	N/A
[24]	Brucellosis	2004–2010	County level	Longitudinal	Characteristics; Risk factors	Livestock density, climate, elevation, and coverage of vegetation
[25]	HFMD	2008.5.1–2009.3.27	County level	Longitudinal	Characteristics; Risk factors	Climate
[26]	Japanese Encephalitis	2002–2010	County level	Longitudinal	Characteristics	N/A
[27]	Tuberculosis	2005–2011	County level	Longitudinal	Characteristics	N/A
[28]	HFMD	2008.5	County level	Cross-sectional	Characteristics; Risk factors	Climate, population density, and socio-economic factors
[29]	HFMD	2008.5	County level	Cross-sectional	Characteristics; Risk factors	Climate, population densities, and economic factors
[30]	HFMD	2008.05.01–2009.03.27	County level	Cross-sectional	Characteristics	N/A
[31]	Hemorrhagic fever	2005–2012	County level	Longitudinal	Characteristics	N/A
[32]	HFMD	2008.5–2013.8	County level	Longitudinal	Characteristics	N/A
[33]	Hepatitis C	2008–2012	City level	Longitudinal	Characteristics	N/A
[34]	Syphilis	2011	County level	Cross-sectional	Characteristics	N/A
[35]	Malaria	2002–2010	County level	Longitudinal	Characteristics	N/A
[36]	H7N9	2013.2–2014.5	City level	Longitudinal	Characteristics	N/A
[37]	H7N9	2013.3–2014.12	Individual level	Cross-sectional	Characteristics; Risk factors	Climate, spatial–temporal factors, and distance to the nearest migration route or habitat of birds
[38]	Dengue	2004–2013	City level	Longitudinal	Characteristics	N/A
[39]	Dengue	2004–2013	City level	Longitudinal	Characteristics	N/A
[40]	Japanese Encephalitis	2013	County level	Cross-sectional	Characteristics	N/A
[41]	Hepatitis B	2005–2014	City level	Longitudinal	Characteristics	N/A
[42]	Anthrax	2005–2012	County level	Longitudinal	Characteristics; Risk factors	Occupational exposure
[43]	Anthrax	2005–2013	County level	Longitudinal	Characteristics; Risk factors	Livestock density, elevation, coverage of vegetation, component of topsoil, and climate
[44]	Rabies	1960–2014	City level	Longitudinal	Characteristics	N/A
[45]	HFMD	2008–2012	County level	Longitudinal	Characteristics	N/A
[46]	Hepatitis C	2008–2013	City level	Longitudinal	Characteristics; Risk factors	Socio-economic factors
[47]	Measles	2005–2014	City level	Longitudinal	Characteristics	N/A
[48]	Dengue	2005–2013	County level	Longitudinal	Characteristics	N/A
[49]	Hemorrhagic fever	2006–2010	City level	Longitudinal	Characteristics	N/A
[50]	Malaria	2005–2014	County level	Longitudinal	Characteristics	N/A
[51]	SARS	2012.11.16–2003.05.21	County level	Longitudinal	Characteristics; Risk factors	Population density and transport accessibility
[52]	Measles	2005 − 2014	City level	Longitudinal	Characteristics	N/A
[53]	Tuberculosis	2005–2014	Individual level	Longitudinal	Characteristics	N/A
[54]	H7N9	2013.2.19–2014.2.16	County level	Longitudinal	Characteristics	N/A
[55]	AIDS	2006–2015	County level	Longitudinal	Characteristics; Risk factors	Population density and socio-economic factors
[7]	Dengue	2005–2017	County level	Longitudinal	Characteristics; Risk factors	Climate and coverage of vegetation
[56]	Syphilis	2010–2015	County level	Longitudinal	Characteristics	N/A
[57]	H7N9	2013.02.19–2017.09.09	County level	Longitudinal	Characteristics; Risk factors	Population density, live-poultry markets density, live-poultry density, and water bird habitat
[58]	HFMD	2009	County level	Longitudinal	Characteristics; Risk factors	Climate and socio-economic factors
[59]	Leptospirosis	2005–2016	County level	Longitudinal	Characteristics	N/A
[60]	Rabies	2005–2013	County level	Longitudinal	Characteristics; Risk factors	Climate, socio-economic factors, and transport accessibility
[61]	Tuberculosis	2005–2015	County level	Longitudinal	Characteristics	N/A
[62]	Tuberculosis	2005–2015	City level	Longitudinal	Characteristics; Risk factors	Climate
[63]	Influenza	2005–2018	City level	Longitudinal	Characteristics; Risk factors	Vaccine number, surveillance protocol, and rate of influenza A (H1N1) pdm09
[64]	H1N1	2009.05.10–2010.04.30	County level	Longitudinal	Characteristics; Risk factors	Transport modes
[65]	H7N9	2013.2.19–2017.9.30	City level	Longitudinal	Characteristics	N/A
[8]	COVID-19	2020.1.24–2020.2.20	City level	Longitudinal	Characteristics; Risk factors	Climate, transport accessibility, population density, and medical facilities
[66]	COVID-19	2019.12.8–2020.3.31	Community level	Cross-sectional	Characteristics	N/A
[67]	Tuberculosis	2013–2018	County level	Cross-sectional	Characteristics	N/A
[68]	COVID-19	2020.1.23–2020.3.23	City level	Longitudinal	Characteristics; Risk factors	Population movement
[69]	COVID-19	2020.1.11–2020.7.31	City level	Longitudinal	Characteristics	N/A
[70]	COVID-19	2020.01.17–2020.03.20	County level	Cross-sectional	Characteristics; Risk factors	Transport accessibility and population density
[71]	COVID-19	2020.01.25–2020.3.13	City level	Cross-sectional	Characteristics; Risk factors	Population movement
[72]	COVID-19	2019.12–2020.03.25	City level	Cross-sectional	Characteristics; Risk factors	Socio-economic factors
[73]	COVID-19	2019.12.1–2020.4.30	City level	Longitudinal	Characteristics; Risk factors	Population movement, climate, air quality and socio-economic factors
[74]	COVID-19	2020.1.10–2020.10.5	City level	Longitudinal	Characteristics	N/A
[75]	COVID-19	2020.1–2020.10	City level	Longitudinal	Characteristics	N/A
[76]	COVID-19	2019.12.2–2020.6.20	Individual level	Cross-sectional	Characteristics	N/A
[77]	H7N9	2013.2.19–2014.3.31	City level	Longitudinal	Characteristics	N/A
[78]	Tuberculosis	2007.1.1–2007.12.31	City level	Longitudinal	Characteristics; Risk factors	Altitude, longitude, climate, education burden, population density, air quality, and economic factors
[79]	Echinococcosis	2018	City level	Cross-sectional	Characteristics	N/A
[80]	Influenza	2004–2017	City level	Longitudinal	Characteristics; Risk factors	Air quality
[81]	H7N9	2013–2017	Individual level	Longitudinal	Characteristics	N/A
[82]	H5N1	2004–2019	City level	Longitudinal	Characteristics	N/A
[83]	COVID-19	2020.1.24–2020.3.5	City level	Longitudinal	Characteristics; Risk factors	Population movement and spatial–temporal factors
[84]	COVID-19	2020.1.24–2020.12.28	City level	Longitudinal	Characteristics; Risk factors	Spatial–temporal factors
[85]	HFMD	2017	City level	Longitudinal	Characteristics; Risk factors	Climate
[86]	HFMD	2017	City level	Longitudinal	Characteristics	N/A

① H7N9: Human infection with H7N9 virus. ② AIDS: Acquired immune deficiency syndrome. ③ H1N1: Influenza A(H1N1) infection. ④ HFMD: Hand, foot and mouth disease. ⑤ H5N1: Human infection with H5N1 virus. ⑥ SARS: Severe acute respiratory syndrome. ⑦ N/A: Not applicable

## Results

### Search results and included studies

#### Search results

**Fig. 1:**
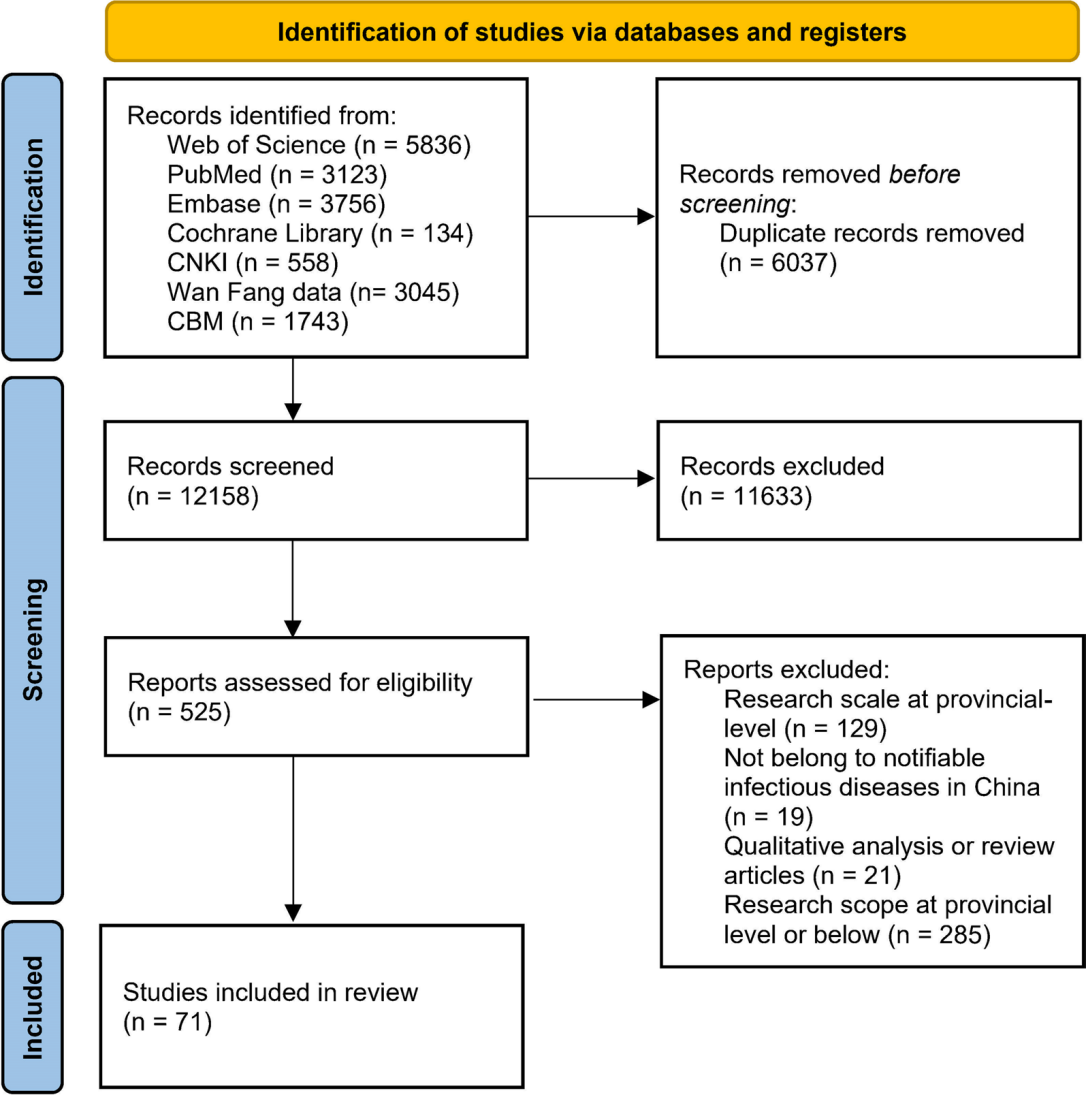
Flow diagram of study selection

A preliminary systematic literature search yielded 18,195 records. After removing duplicates, 12,158 records were retained for screening of the titles and abstracts. Then, 11,633 records were excluded as they did not meet the review eligibility criteria. Of the 525 potentially relevant studies screened in full text, 129 records were at the provincial level, 19 focused on diseases out of the list of notifiable infectious diseases in China, 21 were qualitative analysis or review articles, and 285 focused on specific provincial units or cities rather than the whole territory. The endpoint of the screening process yielded 71 eligible studies (Fig. 1).

#### Characteristics of the studies included

Through a full-text review, we extracted contextual details of the included studies (Table 1). All 71 studies focused on the spatiotemporal characteristics of the incidence of specific infectious diseases. Given that this systematic review focused on small-scale research, the distribution was as follows: 31 studies were analyzed using city level data, 34 studies were analyzed using county level data, and six studies used community or individual data. Among the studies, the longitudinal cohort design (n = 57, 80.28%) was more common than cross-sectional design (n = 14, 19.72%). In addition to the analysis of spatial (or spatiotemporal) characteristics, 29 studies also examined the risk factors (Table 1).

A total of 22 notifiable infectious diseases were included in 71 studies, including 18 class B and 4 class C infectious diseases. Among class B infectious diseases, 13 focused on COVID-19, seven on human infection with H7N9 virus, six on tuberculosis, four on dengue, three on rabies, three on hemorrhagic fever, three on syphilis, two on malaria, two on measles, two on Japanese encephalitis, two on anthrax, two on hepatitis C, and one each on hepatitis B, Acquired immune deficiency syndrome (AIDS), brucellosis, leptospirosis, severe acute respiratory syndrome (SARS), and human infection with H5N1 virus. Among class C infectious diseases, 12 focused on hand, foot and mouth disease (HFMD) and two each on influenza, influenza A(H1N1) virus infection, and echinococcosis (Tables 2). Notably, some studies have also investigated certain subcategories of infectious diseases or their transmission within a specific group, such as fetal syphilis, AIDS among men who have sex with men (MSMs), severe HFMD, SS + tuberculosis, SS − tuberculosis, *P. vivax* malaria, and *P. falciparum* malaria.

**Table 2:** Infectious diseases divided by category and their temporal and spatial trends

Infectious disease class		Infectious diseases		Trend	Hotspots and clusters	References
Class	Number	Items	Number			
Class A	0	–	0	–	–	–
Class B	55	COVID-19	13	The geographical range of COVID-19 transmission expanded but the incidence shrank from 2020.1 to 2020.7	Hubei Province and its surrounding areas (2020.1–2020.3); COVID-19 outbreak in China tended to be decentralized and localized (2020.3–2020.7)	[8, 66, 68, 69, 70, 71, 72, 73, 74, 75, 76, 83, 84]
		H7N9	7	The distribution had shifted from the eastern coastline to more inland areas	Southeast coastline and East China; North China (fifth outbreak)	[4, 24, 57, 59, 77, 81, 87]
		Tuberculosis	6	The geographical range of TB transmission declined from 2005 to 2018. The clustering time of SS + TB was concentrated before 2010 while SS- TB was mainly concentrated after 2010	Northwest and Central China, especially in Xinjiang (2005–2018)	[27, 53, 61, 62, 67, 78]
		Dengue	4	The geographical range of Dengue transmission expanded from 2004 to 2017	South, Southwest, North and East China, moving from the southeast coast to the inland and southwest areas	[7, 38, 39, 48]
		Rabies	3	Rabies incidences experienced M-shaped fluctuations between 1960 and 2014. Since the most recent peak (2007), the number of cases had declined but its geographic range had expanded	South, Central and East China; Expanding to North China	[23, 44, 60]
		Hemorrhagic fever	3	Hemorrhagic fever expanded its geographic limits within China between 1994 and 2012	Northeast, East and South China (1994–1998); Northeast, Northwest, North, and East China (2005–2012), transferring from Northeast and Northwest to East and North China	[18, 31, 49]
		Syphilis	3	The geographical range of syphilis transmission expanded between 2004 and 2011. In 2015, the number of hotspots with prenatal syphilis dropped by more than 65% than in 2010	East, West and Northwest China (2004–2011), especially in Yangtze River delta, Guangxi and expanding from Gansu to Xinjiang; Northeast China (2006, 2008, 2010, 2011), especially in Northern Inner Mongolia	[22, 34, 56]
		Malaria	2	Malaria had been largely eliminated in China from 2002 to 2014	Southwest, East and South China; P. vivax malaria: shifted from the eastern to the western of China; P. falciparum malaria: shifted from the western to the eastern of China	[35, 50]
		Measles	2	The geographical range of Measles transmission decreased from 2005 to 2014	Northwest China, including most of Xinjiang, Tibet, and Western Sichuan (2005–2008); Southern Xinjiang, Tibet, Qinghai, Beijing, Tianjin, central Hebei, and parts of Northeast China (2009–2012); Northwest China, including most of Xinjiang, Tibet, Qinghai, Western Sichuan, and the Pearl River Delta (2013–2014)	[47, 52]
		Japanese encephalitis	2	Japanese encephalitis expanded its geographic limits within China from 2002 to 2010	Southwest China, with an expanding trend to Central China, including Guizhou, Sichuan, Yunnan, Chongqing, Western Hunan, and Southern Shaanxi (2002–2010); Shaanxi-Shanxi-Henan border, Shandong-Hebei border, Sichuan- Chongqing border, and Guizhou (2013)	[26, 40]
		Anthrax	2	Anthrax expanded its geographic limits within China from 2005 to 2013	the border of Southwest and Northwest China, including the Qinghai-Sichuan border and some counties in Gansu and Tibet	[42, 43]
		Hepatitis C	2	The geographical range of Hepatitis C transmission expanded from 2008 to 2013	Northwest and Northeast China, including Gansu, northern Xinjiang, northern Qinghai, western Inner Mongolia, Jilin, southern Heilongjiang, and northern Liaoning	[33, 46]
		Hepatitis B	1	The geographical range of Hepatitis B transmission decreased from 2005 to 2009	Northwest China, including Qinghai, Gansu, Xinjiang, and Western Inner Mongolia; Central China, especially in western Henan	[41]
		AIDS	1	AIDS cases reported among MSM expanded rapidly from 2006 to 2015	East and South China and then spread to Southwest China (2006–2015)	[55]
		Brucellosis	1	The geographical range of Brucellosis transmission expanded from 2004 to 2010	Northeast and Northwest China, and expanding to North China, including Inner Mongolia, Heilongjiang, Shanxi, western Jilin, western Liaoning, northern Shanxi, and northern Xinjiang	[24]
		Leptospirosis	1	The geographical range of Leptospirosis transmission decreased from 2005 to 2015	provincial boundaries in Southwest and East China, including southwest Sichuan, southwest Yunnan, Hubei-Chongqing border, Guizhou-Guangxi border, Fujian-Jiangxi border, and Anhui-Jiangxi-Fujian border	[59]
		SARS	1	SARS has gradually disappeared since its outbreak in 2013	Beijing, the Pearl River Delta, and some other places (2013)	[51]
		H5N1	1	The geographical range of H5N1 transmission decreased from 2004 to 2019	Central China, especially in provincial boundaries Hubei, Hunan, Anhui, and Jiangxi (2004); Urumqi and its surrounding cities (2015); Northwest China, such as Xinjiang, Tibet, and Qinghai Province (2006–2012, 2018) Parts of Yunnan and Guizhou Province (2013–2016); Northeast China (2017, 2019)	[82]
Class C	16	HFMD	12	The geographical range of HFMD transmission expanded from 2008 to 2013	North, East, and South China, with scope in South China expanding (including Beijing, Tianjin, Hebei, and northern Shanxi) and that in North China narrowing (Guangdong, Guangxi, and Hainan)	[19, 20, 21, 25, 28, 29, 30, 32, 45, 58, 85, 86]
		Influenza	2	The geographical range of Influenza transmission expanded	Influenza was distributed all over China	[63, 80]
		H1N1	1	H1N1 had gradually disappeared since its outbreak in 2009	Central, East, and South China, including the Pearl River Delta, central Hebei, and northern Hubei	[64]
		Echinococcosis	1	No clear trend	Southwest and Central China, Qinghai-Tibet Plateau area	[79]

① H7N9: Human infection with H7N9 virus. ② AIDS: Acquired immune deficiency syndrome. ③ H1N1: Influenza A(H1N1) infection. ④ HFMD: Hand, foot and mouth disease. ⑤ MSM: Men who have sex with men. ⑥ H5N1: Human infection with H5N1 virus. ⑦ SARS: Severe acute respiratory syndrome

**Fig. 2:**
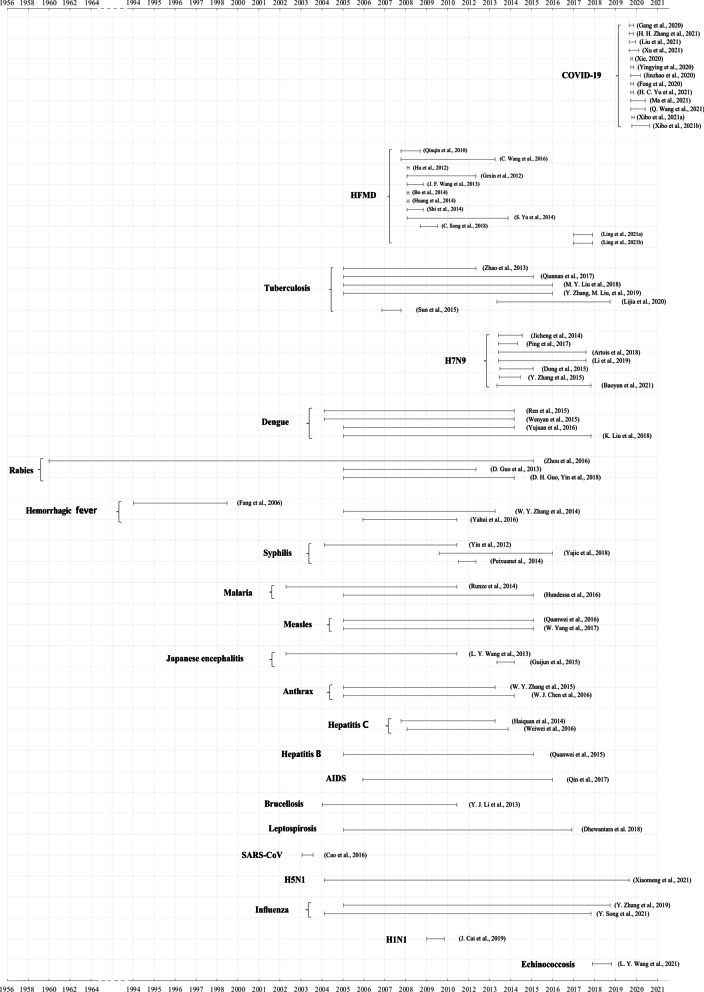
Research durations of studies included

All studies were published after 2006, of which 66 studies used data for a certain period between 2004 and 2021, mostly due to the implementation of the direct network reporting system of legal infectious diseases in China. The remaining five literature used data earlier than 2004, and they studied infectious diseases including rabies, hemorrhagic fever, Japanese encephalitis, SARS-Cov and malaria (Fig. 2). There are some studies of short durations, with COVID-19 outbreaks close to the present, and short durations of SARS and influenza A(H1N1) virus infection outbreaks.

### Spatiotemporal methods

Among the included studies, a variety of spatial and spatiotemporal methods were employed to visualize risk patterns, explore spatial clusters, and model determinants of disease transmission. These methods can be divided into four categories: visualization, cluster detection, spatial exploration, and spatiotemporal modelling.

Fifty-eight articles (81.69%) presented or referred to the method of visualizing case distributions to describe the spatiotemporal epidemiology of notifiable infectious diseases in China. The most frequently used method was rate maps (n = 57), with data usually aggregated according to administrative boundaries. Kernel density maps were used in four studies. Other methods included excess hazard maps (n = 2), spatially smoothed percentile map (n = 1), continuous distribution map (n = 1), and relative risk map (n = 1).

The use of at least one cluster detection method was reported in 54 studies (76.06%), of which the most frequently used method was Moran’s I statistic (n = 41), followed by Kulldorff space–time scan statistic (n = 26), LISA cluster maps (n = 24), and Getis-Ord Gi* statistic (n = 18). The K-nearest neighbor test, standard deviation elliptical analysis, and optimized/emerging hotspot analysis were applied twice. The average nearest neighbor distance method and density-based spatial clustering of applications with noise were used in the same study.

A range of other methods based on the spatial exploration of cases was also identified. Six methods of spatial exploration, including the hierarchical cluster analysis, Bayesian hierarchical model, Spearman rank correlation analysis method, empirical orthogonal function analysis, and Fréchet distance approach, were used in different studies.

Spatiotemporal modelling was applied in 29 studies (40.85%) to explore risk factors. The geographically weighted regression model (GWR) was the most frequently used method, utilized in seven studies. Poisson regression (n = 6), the geographical detector method (n = 4), the Bayesian spatial model (n = 3), and linear regression (n = 3) were ranked successively. The generalized linear model (GLM), Lasso regression, boosted regression trees (BRT), and spatial Durbin model (SDM) were used more than once (Table 3).

**Table 3:** Spatiotemporal methods used in studies included

Category	Number	Method	Number	References
Visualization	58	Rate map	57	[7, 8, 18, 19, 20, 22, 24, 25, 26, 28, 29, 30, 31, 35, 36, 37, 38, 39, 41, 42, 44, 45, 47, 48, 49, 50, 51, 52, 53, 54, 55, 57, 58, 59, 60, 61, 62, 63, 64, 65, 66, 67, 68, 70, 71, 72, 73, 74, 75, 76, 77, 78, 79, 80, 84, 85, 86]
		Kernel density map	4	[23, 45, 53, 66]
		Excess hazard map	2	[18, 58]
		Spatially smoothed percentile map	1	[18]
		Continuous distribution map	1	[18]
		Relative risk map	1	[67]
Cluster (Hotspot) Detection	54	Moran’s I statistic	41	[7, 8, 18, 21, 22, 25, 26, 27, 29, 31, 33, 34, 36, 38, 39, 40, 41, 45, 47, 49, 50, 54, 55, 56, 59, 62, 65, 66, 67, 68, 69, 70, 71, 73, 75, 79, 80, 82, 83, 85, 86]
		Kulldorff space–time scan statistic	26	[7, 18, 26, 27, 31, 32, 33, 35, 40, 42, 43, 45, 46, 49, 50, 55, 61, 65, 67, 69, 73, 74, 75, 76, 79, 83]
		LISA cluster map	24	[7, 8, 18, 21, 22, 26, 29, 33, 34, 38, 39, 40, 41, 45, 47, 49, 55, 56, 59, 69, 70, 73, 74, 75]
		Getis-Ord Gi* statistic	18	[21, 33, 34, 36, 38, 39, 49, 53, 56, 62, 65, 66, 68, 73, 74, 75, 77, 80]
		K-nearest neighbor test	2	[18, 21]
		Standard deviation elliptical analysis	2	[66, 81]
		Optimized/emerging hot spot analysis	2	[83, 84]
		Average nearest neighbor distance method	1	[23]
		Density-based spatial clustering of applications with noise	1	[23]
Spatial exploration	10	Hierarchical cluster analysis	3	[52, 82, 86]
		Bayesian hierarchical model	2	[54, 58]
		Spatial Markov chain model	2	[83, 84]
		Spearman rank correlation analysis method	1	[75]
		Empirical orthogonal function analysis	1	[30]
		Fréchet distance approach	1	[25]
Spatial/Spatio-temporal modelling	29	GWR	7	[20, 46, 62, 68, 72, 73, 78]
		Poisson regression	6	[24, 42, 46, 61, 63, 72]
		Geographical detector method	4	[8, 25, 29, 85]
		Bayesian spatial model	3	[22, 51, 63]
		Linear regression	3	[55, 64, 71]
		Lasso regression	2	[58, 73]
		GLM	2	[57, 72]
		BRT	2	[43, 57]
		SDM	2	[83, 84]
		GAM	1	[70]
		Logistic regression	1	[37]
		Granger causality analysis	1	[24]
		Cochran-Armitage trend test	1	[55]
		Kruskal–Wallis test	1	[42]
		Ecological niche model	1	[7]
		GMM	1	[60]

① GWR: Geographically weighted regression model. ② GLM: Generalized linear model. ③ BRT: Boosted regression trees. ④ GAM: Generalized additive model. ⑤ SDM: Spatial dubin model. ⑥ GMM: Gaussian mixed model

### Spatiotemporal distribution characteristics

**Fig. 3:**
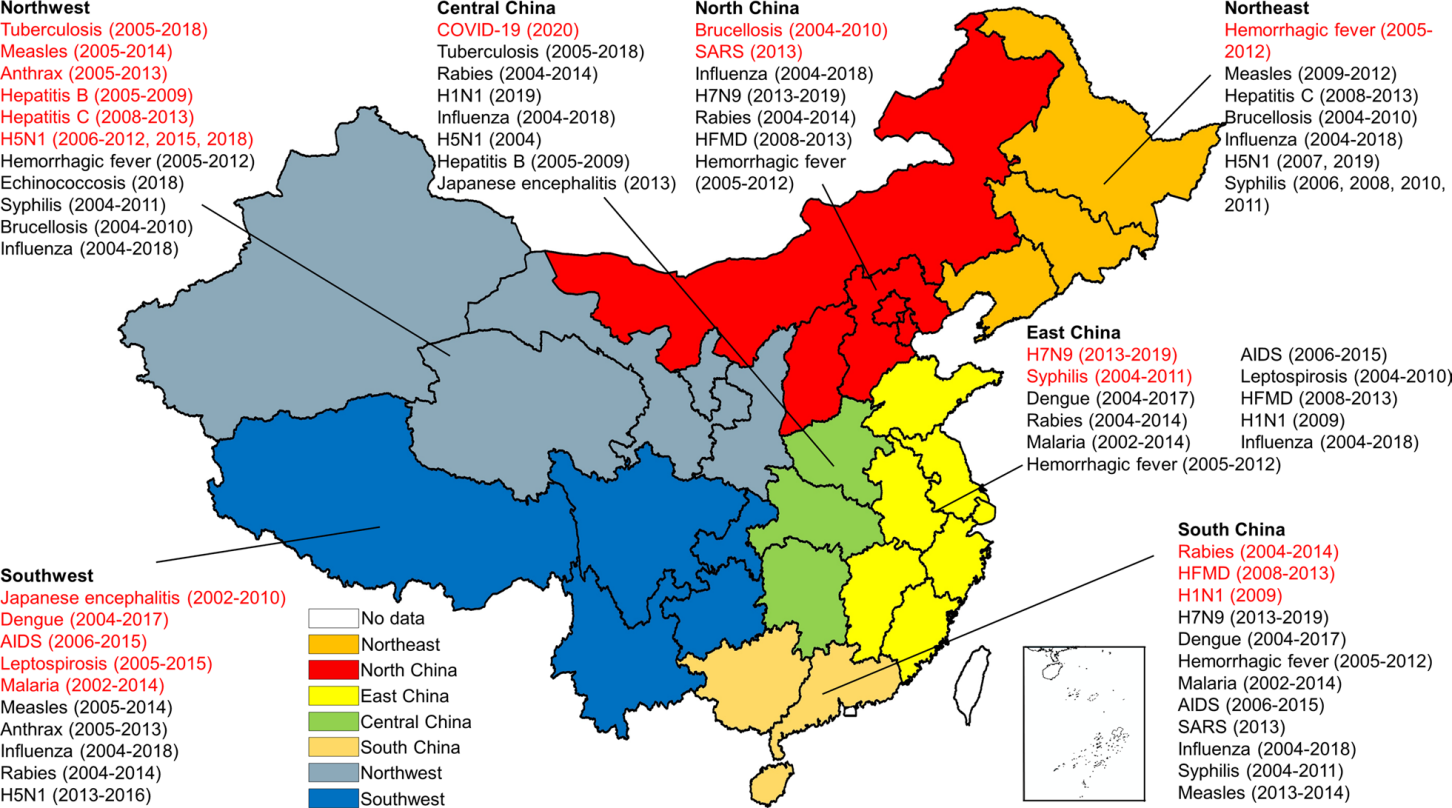
Geographical distribution map of notifiable infectious diseases in China. The clusters of 22 notifiable infectious diseases included in this review were distributed across the indicated region (based on the seven geographical divisions of China) and had been marked in the map. If there are clusters (but not the main cluster) of the infectious disease in this region, the name and periods of existence presented in black; if there is the main cluster of the infectious disease in this region, the name and periods of existence presented in red

Spatiotemporal analysis focused on 22 infectious diseases that accounted for nearly half of all 45 notifiable infectious diseases; and the characteristics of these infectious diseases was shown in Table 2. We counted the high-risk spatiotemporal clusters of infectious diseases that need attention in each region based on the seven geographical divisions of China (Fig. 3). The results showed that clusters of twelve infectious diseases existed in South China, which had the most infectious disease clusters. East, Southwest, and Northwest China each had eleven infectious diseases’ cluster area. Central China had eight. Northeast and North China each had seven.

#### Infectious diseases which mainly clustered in South China

Specifically, the main cluster area of infectious diseases was different. Rabies, HFMD, and influenza A(H1N1) virus infection mainly clustered in South China. The incidence of rabies experienced M-shaped fluctuations between 1960 and 2014. Since the most recent peak (2007), the number of cases has declined, but the geographic range has expanded. The high-value clusters were mostly located in South, Central, and East China, and expanded to North China. From 2008 to 2013, the cluster scope of HFMD in South China expanded with the shrinkage of North China. Influenza A(H1N1) virus infection is widely distributed in densely populated areas of China, especially in the Pearl River Delta, central Hefei, and northern Hubei (shown in Table 2).

#### Infectious diseases which mainly clustered in East China

Human infection with H7N9 virus and syphilis mainly clustered in East China. The high-value clusters of human infection with H7N9 virus were mainly distributed along the eastern and southeastern coastlines of China, and it had recently shifted to more inland areas. The distribution of syphilis cases expanded from 2004 to 2011; areas with high-high clusters were mainly located in the East, Northeast, and Southwest China, and expanding to Northwest China (shown in Table 2).

#### Infectious diseases which mainly clustered in Southwest China

Dengue, malaria, Japanese encephalitis, AIDS, and leptospirosis mainly clustered in Southwest China. Dengue cases increased between 2004 and 2017, with primary clusters mainly distributed along the southeast coastal areas and southwest border regions of China and transmitted to the inland and southwest areas. A great advance had been made in the control of malaria in China, but with high-value clusters mainly located in Southwest, East, and South China from 2002 to 2014. Japanese encephalitis expanded its high-high cluster limits from Guizhou, Sichuan, Yunnan, and Chongqing to central China, such as the Western Hunan and Shaanxi-Shanxi-Henan borders. AIDS cases reported among MSM increased rapidly from 2006 to 2015, with the clusters expanding from East and South China to Southwest China. High-high clusters of leptospirosis are usually located in inter-provincial border areas (shown in Table 2).

#### Infectious diseases which mainly clustered in Northwest China

Tuberculosis, measles, anthrax, hepatitis B, hepatitis C, and human infection with H5N1 virus mainly clustered in Northwest China. The geographical range of tuberculosis transmission decreased from 2005 to 2018. Most of the primary clusters were located in Northwest and Central China, especially in the Xinjiang province. The clustering time of SS + tuberculosis was concentrated before 2010, whereas that of SS − tuberculosis was mainly concentrated after 2010. It is worth noting that the primary clusters of fetal syphilis decreased by more than 65% in 2015 compared to 2010. Measles had clusters in most of Xinjiang, Tibet, and Qinghai, with a tendency to decrease from 2005 to 2014. The primary clusters of Anthrax covered the Qinghai-Sichuan border and some counties in Gansu and Tibet. Hepatitis B and hepatitis C both had high-value clusters in Qinghai, Gansu, Xinjiang, and Inner Mongolia, while there was a cluster of hepatitis B in western Henan and hepatitis C in some cities in Northeast China. Human infection with H5N1 virus is a subtype of highly pathogenic avian influenza that has gradually disappeared since its outbreak in 2009 (shown in Table 2).

#### Infectious diseases which mainly clustered in Central China

COVID-19 mainly clustered in Central China. In 2020, the COVID-19 outbroke and tended to be decentralized and localized, and incidences were scattered across China; however, the number dropped dramatically, with the primary clusters located in Hubei Province and its surrounding areas (shown in Table 2).

#### Infectious diseases which mainly clustered in Northeast China

Hemorrhagic fever mainly clustered in Northeast China. The geographic limits of hemorrhagic fever expanded in China from 1994 to 2012. Areas with high incidence were mainly concentrated in Northeast and Northwest China, with a tendency toward East and North China (shown in Table 2).

#### Infectious diseases which mainly clustered in North China

Brucellosis and SARS mainly clustered in North China. Areas with a high incidence of brucellosis were mainly located in Northeast and Northwest China, where animal husbandry was developed, with a tendency to expand to North China. SARS mainly occurred in Beijing, Pearl River Delta (shown in Table 2).

### Risk factors of notifiable infectious diseases in China

Twenty-nine articles further explored the risk factors that influenced the spread of infectious diseases. Of the 22 infectious diseases analyzed, 14 were included in the risk factor analysis. According to the results, potential risk factors for infectious diseases mainly include meteorological, socio-economic, population, and spatial–temporal factors, and public health conditions. Among these factors, climate (n = 16), socioeconomic factors (n = 9), population density (n = 8), transport accessibility (n = 5), population movement (n = 4), and air pollution (n = 4) had a broad impact on various infectious diseases (Additional file 1: Supplemental file 2).

## Discussion

In this systematic review, we included 71 studies on the small-scale spatiotemporal epidemiology of notifiable infectious diseases in China, involving 22 infectious diseases. Specifically, 13 studies on COVID-19, 12 on HFMD, seven on human infection with H7N9 virus, and six studies on tuberculosis, which were widely distributed in China during certain periods and attracted public concern, were included. It indicated that researchers have paid considerable attention to emerging infectious diseases and some infectious diseases that impose a heavy burden in China. Small-scale spatiotemporal epidemiology analysis has enhanced the understanding of the patterns and determinants of notifiable infectious diseases.

Spatial methods have been widely used in the spatiotemporal analysis of notifiable infectious diseases in China, especially for visualization and cluster detection. Although the rate map was the most commonly used method, a wide range of novel visualization techniques were applied, including kernel density maps, excess hazard maps, and spatially smoothed percentile maps. For the cluster detection methods, spatial autocorrelation analysis based on Moran’s I statistics was applied most frequently in 41 studies, in which approximately half of the studies were presented visually using the LISA cluster map. The Kulldorff space–time scan statistic and Getis-Ord Gi* statistic were the most commonly used cluster detection methods. The results of cluster detection are sensitive to changes in the boundaries into which they are grouped [87]; thus, analysis on a small scale, such as city or county level, is much better than at a province level, helping identify high-risk areas more accurately. Nevertheless, assessing the presence of this effect should be a priority for future studies using aggregated data from spatiotemporal epidemiology studies [13]. Notably, in studies that incorporated more than one cluster detection method, areas identified as hotspots were not identical, with the extent of agreement between alternative methods being highly variable. As a result, an accumulating body of research suggests the use of multiple clustering detection methods and requires their overlap to represent truly high-risk areas [88, 89]. 29 studies were analyzed using spatiotemporal modelling to explore the risk factors influencing the spread of infectious diseases. The geographically weighted regression model, Poisson regression, and the geographical detector method were the three most commonly used methods. Accounting for spatial correlation could improve model fit, as confirmed in the studies included in this systematic review, because conventional regression models assume spatial independence of model residuals and ignore the potential presence of spatial autocorrelation [24, 62, 77]. Different spatial analysis methods own different advantages in visulation, cluster detection and risk factor exploration. Researchers choose spatial analysis methods based on data availability and research objectives, sometimes use different methods for sensitivity analysis. For instance, Moran’s I is one of the most common spatial autocorrelation indicators, which has the unique advantage of detecting four types of spatial clusters (high-high, low-low, Low–high, high-low). Kulldorff’sspace-time scan statistic is defined by a circular window with a geographic base and with height corresponding to time, thus the statistic may be used for either a single retrospective analysis, using historic data, or for time-periodic prospective surveillance. GWR is applied under the assumption that the strength and direction of the relationship between a dependent variable and its predictors may be modified by contextual factors, while limitations of GWR include problems of multicollinearity and the approaches to calculating goodness of fit statistics.

While our review focused on methodological issues, some consistent observations about the characteristics of disease distribution also received attention. First, the trend of notifiable infectious diseases in China has presented certain patterns. The incidence and coverage of tuberculosis, human infection with H7N9 virus, rabies, hemorrhagic fever, malaria, measles, Japanese encephalitis, leptospirosis, influenza A(H1N1) virus infection, and SARS have decreased significantly in the past decade, of which, malaria has almost been eliminated [26, 31, 50, 51, 52, 59, 60, 62, 64]; this may be attributed to the extensive public disease control and ecological and health improvement in China [52]. However, dengue, syphilis, hepatitis C, AIDS, brucellosis, HFMD, and influenza showed the opposite trend because of the rapid development of tourism and the considerable increase in the migrant population in recent years [7, 11, 22, 55, 58, 80, 90]. Through spatiotemporal analyses of the included studies, it was found that the primary clusters of dengue, hemorrhagic fever, syphilis, Japanese encephalitis, anthrax, hepatitis C, brucellosis, HFMD, and human infection with H7N9 virus were expanding [11, 22, 24, 26, 31, 43, 65, 80]. These diseases have a common tendency to spread to the west and inland areas, which was explained by the included studies, as the phenomenon might be related to the increase in population mobility and improvement of transportation infrastructure that has made less developed and inland areas more closely connected with the outside world [64, 91].

Second, through this systematic review, two main trends could be summarized to describe the regional transmission patterns of notifiable infectious diseases in China. Natural focal infectious diseases and vector-borne diseases spread from specific areas, such as hot and humid areas, plateaus, and grasslands, to other areas. One possible explanation is that global warming and climate anomalies lead to an expansion in the activity range of virus hosts [24, 92]. On the other hand, clusters of infectious diseases, especially respiratory and intestinal infectious diseases, expanded to poor mountainous areas and minority regions in the western China. The reason might be the rapid urbanization process, more convenient public transport, connecting the latter two foci, and likely facilitating transmission across regions [24, 64]. In particular, the seasonal return of migrant workers to their hometowns might have facilitated the reintroduction of infectious diseases to rural communities in mountainous areas and minority regions, which are economically underdeveloped and lack health resources [52]. These reasons have greatly increased the epidemic prevention pressure on local governments.

Risk factors of infectious diseases have been well explored. In almost all reviewed studies, meteorological factors, socioeconomic factors, population factors, and potential contact with livestock (if zoonotic disease) were documented to be significantly related to the incidence rates of notifiable infectious diseases in China, although it is difficult to rule out publication bias favoring studies with positive findings. This result is consistent with our observation that most of the diseases were gathered in the area east of the Hu Huanyong Line, including rabies, dengue, Japanese encephalitis, leptospirosis, and influenza A(H1N1) virus infection, which were contributed by the warm and humid climate, relatively advanced economy, high population density, and frequent population movements in the area east of the Hu Huanyong Line. However, factors which have a notable impact on the spread of the disease, such as medical facilities, exposure to livestock or poultry, and government interventions, were rarely considered. Future studies could incorporate these into models as independent variables of interest or control variables. In addition, small-area analyses enable researchers to fully explore the causal relationship between diseases and their potential risk factors, making it more statically unbiased [93]. Using aggregate data at the province or country level often leads to a modifiable areal unit problem (MAUP), which refers to the fact that aggregating data into larger sizes or geographical units for spatial analysis can cause many problems, including accuracy, scale, quality (possible bias), and confounding factors [94]. One of the best ways to solve the MAUP is to use data that provide detailed information about spatial units at the small-area level; if failed to do so, it is difficult to guarantee the reliability of the results [95].

Over the past four decades, China has experienced a large-scale modification in the landscape due to industrialization and urbanization, which has possibly led to rapid changes in the regional transmission pattern of infectious diseases in China [5, 96]. Although the number of cases in China has declined steadily over the past few years, most infectious diseases have paradoxically occurred in a wider geographical area, which ought to draw more attention from policymakers. Given the limited resources available, targeted efforts in different areas are required. First, local governments should pay attention to the temporal and spatial trends in the expansion of infectious diseases and prepare for epidemic prevention in advance. In cluster areas, sufficient funding is needed to improve sanitation and sanitation infrastructure [97]. Second, comprehensive control measures, including political commitment to control programs, inter-sectoral coordination, sensitive surveillance systems, accessibility to modern vaccines, awareness education, and cooperation should be strengthened in China [98]. Third, health education and promotion campaigns among both residents and physicians are priorities in atypical areas [99].

There are still a few limitations in the small-scale spatiotemporal epidemiology of notifiable infectious diseases in China. First, some diseases are widely distributed in China, but their spatiotemporal epidemiology has not been studied on a small scale, especially infectious diseases in class C. In addition, previous studies must be updated owing to the earlier production time. Second, ArcGIS, SaTScan, and Geoda are still the most commonly used software, limiting the application of new spatiotemporal methods. There is scope for the development of new tools for the analysis and visualization of spatial data. Third, it is important to systematically summarize the temporal and spatial characteristics of notifiable infectious diseases in China on a small scale. However, for the current research status, there are still over half of notifiable infectious diseases in China that have not been studied in small-scale spatiotemporal epidemiology, including gonorrhea and scarlet fever. For the included studies, the research time interval overlapped significantly. Applying spatiotemporal epidemiological methods to study infectious diseases can inform decision-makers and other stakeholders regarding where and when to improve targeted response measures to mitigate further transmission. Fourth, the data of most studies included were compiled from the CISDCP, which could indicate that the data were underreported. Passive surveillance data could cause some cases to go unreported because of their milder clinical symptoms, or some could be delayed in reporting because of delayed diagnosis in rural settings. Researchers should increase the number of studies in which data are collected through active surveillance.

## Conclusion

In summary, small-scale spatiotemporal epidemiology has made great progress in the past 20 years and been widely applied in improving the understanding of notifiable infectious diseases in China, including the distribution, clusters, trends, risk factors, and the mechanisms driving the local epidemiology. As data types and sources become increasingly rich and complex, we encourage researchers to use the latest publicly available data and to focus on infectious diseases that have not yet been studied in China, and more attention should be paid to systematically exploring the factors that contribute to spatial differences in infectious diseases.

## Supplementary Information

**Additional file 1: Supplemental files 1.** Classification of Notifiable Infectious Diseases in China. **Supplemental files 2.** Risk factors Influencing spatio-temporal epidemiology of notifiable infectious diseases in China. **Supplemental files 3.** The Search Strategy.

## Data Availability

The datasets generated during and analyzed during the current study are available from the corresponding author on reasonable request.

## Abbreviations

COVID-19: Coronavirus disease 2019
CISDCP: China Information System for Disease Control and Prevention
MSM: Men who have sex with men
MAUP: Modifiable areal unit problem
SARS: Severe acute respiratory syndrome
AIDS: Acquired immune deficiency syndrome
HFMD: Hand, foot and mouth disease
H7N9: Human infection with H7N9 virus
H5N1: Human infection with H5N1 virus
H1N1: Influenza A(H1N1) virus infection
