# Supplementary material for: Small-scale spatiotemporal epidemiology of notifiable infectious diseases in China: a systematic review
Source: BMC Infect Dis. 2022 Sep 5;22:723. doi: 10.1186/s12879-022-07669-9 (PMC9442567; doi:10.1186/s12879-022-07669-9)
Supplement: Supplementary file 1 — Additional file 1: Supplemental files 1. Classification of Notifiable Infectious Diseases in China. Supplemental files 2. Risk factors Influencing spatio-temporal epidemiology of notifiable infectious diseases in China. Supplemental files 3. The Search Strategy. [file 12879_2022_7669_MOESM1_ESM.docx]

**Supplemental files 1.** Classification of Notifiable Infectious Diseases in China

| Classification | Items |
| --- | --- |
| Classes | Class A: Plague, Cholera  Class B: Severe acute respiratory syndrome (SARS), Acquired immune deficiency syndrome (AIDS), Viral hepatitis (including A, B, C, D, E and unspecified), Poliomyelitis, Human infections of highly pathogenic avian influenza, human infection of H7N9 virus, Measles, Epidemic hemorrhagic fever(EHF), Rabies, Epidemic Encephalitis B, Dengue, Anthrax, Bacterial and amoebic dysentery, Tuberculosis, Typhoid & paratyphoid (typhoid and paratyphoid are counted as a single disease type in the law) , Epidemic (meningococcal) meningitis, Pertussis, Diphtheria, Neonatal tetanus, Scarlet fever, Brucellosis, Gonorrhea, Syphilis, Leptospirosis, Schistosomiasis, Malaria, Corona Virus Disease 2019 (COVID-19)  Class C: Influenza, Mumps, Rubella, Acute hemorrhagic conjunctivitis (AHC), Leprosy, Typhus, Leishmaniasis, Echinococcosis, Filariasis, Other infectious diarrheal diseases, Hand, foot and mouth disease (HFMD) |
| Transmission routes | Direct contact/fecal-oral transmitted diseases (cholera, Hepatitis A, Hepatitis E, Other hepatitis, poliomyelitis, Bacillary and Amebic dysentery, Typhoid and paratyphoid, Acute hemorrhagic conjunctivitis, Hand, foot and mouse disease); Respiratory transmitted diseases (SARS, Mumps, Tuberculosis, Influenza, Poliomyelitis, Rubella, Lepriasis); Zoonotic/vectorborne diseases (Plague, Avian influenza virus, Epidemic hemorrhagic fever, Rabies, Epidemic encephalitis B, Dengue fever, Brucellosis, Leptospirosis, Schistosomiasis, Malaria, Influenza A(H1N1) virus infection, Human infection with H5N1 virus, Human infection of H7N9 virus, Epidemic and endemic typhus, Kala-azar, Echinococcosis, Filariasis); Blood and sexual transmitted type (AIDS, Hepatitis B, Hepatitis E, Gonorrhea, Syphilis); other infectious diseases (neonatal tetanus) |
| Pathogens | Bacterial diseases (Plague, Cholera, Anthrax, Bacterial dysentery, Tuberculosis, Typhoid fever and paratyphoid fever, Poliomyelitis, Pertussis, Diphtheria, Neonatal tetanus, Scarlet fever, Brucellosis, Gonorrhea, Syphilis, Lepriasis, Epidemic and endemic typhus, Leptospirosis); Virosis (SARS, AIDS, Viral hepatitis, Poliomyelitis, Avian influenza virus, Mumps, Epidemic hemorrhagic fever, Rabies, Epidemic encephalitis B, Dengue fever, Human infections with avian influenza A, influenza, Rubella, Acute hemorrhagic conjunctivitis, Hand, foot and mouse disease); Parasitic diseases (Amebic dysentery, Schistosomiasis, Malaria, Filariasis, Echinococcosis, Kalaazar) |

**Supplemental files 2.** Risk factors Influencing spatio-temporal epidemiology of notifiable infectious diseases in China (continuing on next page)

| Infectious Diseases | Climate | Socio-economic factors | Population density | Transport accessibility | Air quality | Population movement | Medical facilities | Spatial-temporal factors | Live-poultry markets density |
| --- | --- | --- | --- | --- | --- | --- | --- | --- | --- |
| COVID-19 | ✓ | ✓ | ✓ | ✓ | ✓ | ✓ | ✓ | ✓ |  |
| H7N9 | ✓ |  | ✓ |  |  |  |  | ✓ | ✓ |
| Tuberculosis | ✓ | ✓ | ✓ |  | ✓ |  |  |  |  |
| Dengue | ✓ |  |  |  |  |  |  |  |  |
| Rabies | ✓ | ✓ |  | ✓ |  |  |  |  |  |
| Hemorrhagic fever |  |  |  |  |  |  |  |  |  |
| Syphilis |  |  |  |  |  |  |  |  |  |
| Malaria |  |  |  |  |  |  |  |  |  |
| Measles |  |  |  |  |  |  |  |  |  |
| Japanese encephalitis |  |  |  |  |  |  |  |  |  |
| Anthrax | ✓ |  |  |  |  |  |  |  |  |
| Hepatitis C |  | ✓ |  |  |  |  |  |  |  |
| Hepatitis B |  |  |  |  |  |  |  |  |  |
| AIDS |  | ✓ | ✓ |  |  |  |  |  |  |
| Brucellosis | ✓ |  |  |  |  |  |  |  |  |
| Leptospirosis |  |  |  |  |  |  |  |  |  |
| SARS |  |  | ✓ | ✓ |  |  |  |  |  |
| H5N1 |  |  |  |  |  |  |  |  |  |
| HFMD | ✓ | ✓ | ✓ |  |  |  |  |  |  |
| Influenza |  |  |  |  | ✓ |  |  |  |  |
| H1N1 |  |  |  | ✓ |  |  |  |  |  |
| Echinococcosis |  |  |  |  |  |  |  |  |  |
| Total number | 8 | 6 | 6 | 4 | 3 | 1 | 1 | 2 | 1 |

**Notes:** ①H7N9: Human infection with H7N9 virus. ②AIDS: Acquired immune deficiency syndrome. ③H1N1: Influenza A(H1N1) infection. ④HFMD: Hand, foot and mouth disease. ⑤H5N1: Human infection with H5N1 virus. ⑥SARS: Severe acute respiratory syndrome.

**Supplemental files 2.** Risk factors Influencing spatio-temporal epidemiology of notifiable infectious diseases in China (Continued from previous page)

| Infectious Diseases | Live-poultry density | Livestock density | Coverage of vegetation | Altitude | Occupational exposure | Elevation | Longitude | Vaccine number | Other factors |
| --- | --- | --- | --- | --- | --- | --- | --- | --- | --- |
| COVID-19 |  |  |  |  |  |  |  |  |  |
| H7N9 | ✓ |  |  |  |  |  |  |  | distance to the neare4st migration route or habitat of birds |
| Tuberculosis |  |  |  | ✓ |  |  | ✓ |  | education burden |
| Dengue |  |  | ✓ |  |  |  |  |  |  |
| Rabies |  |  |  |  |  |  |  |  |  |
| Hemorrhagic fever |  |  |  |  |  |  |  |  |  |
| Syphilis |  |  |  |  |  |  |  |  |  |
| Malaria |  |  |  |  |  |  |  |  |  |
| Measles |  |  |  |  |  |  |  |  |  |
| Japanese encephalitis |  |  |  |  |  |  |  |  |  |
| Anthrax |  | ✓ | ✓ |  | ✓ | ✓ |  |  | component of topsoil |
| Hepatitis C |  |  |  |  |  |  |  |  |  |
| Hepatitis B |  |  |  |  |  |  |  |  |  |
| AIDS |  |  |  |  |  |  |  |  |  |
| Brucellosis |  | ✓ | ✓ |  |  | ✓ |  |  |  |
| Leptospirosis |  |  |  |  |  |  |  |  |  |
| SARS |  |  |  |  |  |  |  |  |  |
| H5N1 |  |  |  |  |  |  |  |  |  |
| HFMD |  |  |  |  |  |  |  |  |  |
| Influenza |  |  |  |  |  |  |  | ✓ | surveillance protocol, rate of influenza A (H1N1) pdm09 |
| H1N1 |  |  |  |  |  |  |  |  |  |
| Echinococcosis |  |  |  |  |  |  |  |  |  |
| Total number | 1 | 2 | 3 | 1 | 1 | 2 | 1 | 1 | 4 |

**Notes:** ①H7N9: Human infection with H7N9 virus. ②AIDS: Acquired immune deficiency syndrome. ③H1N1: Influenza A(H1N1) infection. ④HFMD: Hand, foot and mouth disease. ⑤H5N1: Human infection with H5N1 virus. ⑥SARS: Severe acute respiratory syndrome.

**Supplemental files 3.** The Search Strategy

**Keyword**

- China/ Chinese
- space/ spatial/ space-time/ spatio-temporal/ geographic/ geographical/ GIS/ mapping
- infectious diseases/ communicable diseases/ infection/ emerging infectious diseases/ epidemic/ bacterial infections/ virus diseases/ parasitic diseases/ mycoses/ central nervous system infections/ zoonoses/ zoonosis/ vaccine preventable diseases/ sexually transmitted diseases/ std/ sexually transmitted agents/ quarantinable diseases/ gastrointestinal diseases/ vector-borne diseases
- plague/ cholera/ severe acute respiratory syndrome/ SARS/ acquired immune deficiency syndrome/ AIDS/ HIV/ hepatitis/ hepatitis A/ hepatitis B /hepatitis C/ hepatitis D/ hepatitis E/ unspecified hepatitis/ other hepatitis/ poliomyelitis/ human infections of highly pathogenic avian influenza/ Influenza A/ H1N1/ H5N1/ H7N9/ measles/ epidemic hemorrhagic fever/ EHF/ rabies/ epidemic encephalitis B/ EEB/ Japanese encephalitis/ dengue/ anthrax/ dysentery/ tuberculosis/ typhoid/ paratyphoid/ meningitis/ pertussis/ diphtheria/ neonatal tetanus/ scarlet fever/ brucellosis/ gonorrhea/ syphilis/ leptospirosis/ schistosomiasis/ malaria/ corona virus disease 2019 / COVID-19/ influenza/ mumps/ rubella/ acute hemorrhagic conjunctivitis/ AHC/ leprosy/ typhus/ kala azar/ leishmaniasis/ echinococcosis/ filariasis/ infectious diarrhea / Hand, foot and mouth disease/ HFMD
- 中国、中国的、我国、我国的
- 空间、时空、地理
- 传染病、传染性疾病、流行病、疫情
- 鼠疫、霍乱、新型冠状病毒肺炎、新冠肺炎、传染性非典型肺炎、非典、艾滋病、病毒性肝炎、脊髓灰质炎、小儿麻痹症、人感染高致病性禽流感、禽流感、甲流、H1N1、H5N1、H7N9、猪流感、麻疹、流行性出血热、出血热、狂犬病、流行性乙型脑炎、乙脑、登革热、炭疽、细菌性痢疾和阿米巴性痢疾、痢疾、肺结核、伤寒和副伤寒、伤寒、流行性脑脊髓膜炎、流脑、百日咳、白喉、新生儿破伤风、猩红热、布鲁氏菌病、布病、淋病、梅毒、钩端螺旋体病、钩体病、血吸虫病、疟疾、流行性感冒、流感、流行性腮腺炎、腮腺炎、风疹、急性出血性结膜炎、结膜炎、麻风病、斑疹伤寒、黑热病、包虫病、丝虫病、感染性腹泻、手足口病

**Search strategy**

**Database 1: Web of Science Core collection**

| No. | Query | Results |
| --- | --- | --- |
| #1 | TS=(China) OR TS=(Chinese) | 1,096,820 |
| #2 | TS=(“space”) OR TS=(spatial) OR TS=(space-time) OR TS=(spatio-temporal) OR TS=(geographic) OR TS=(geographical) OR TS=(GIS) OR TS=(“mapping”) | 3,460,539 |
| #3 | TS=(epidemic) OR TS=(infectious-diseases) OR TS=(communicable-disease) OR TS=(infection) OR TS=(emerging-infectious-diseases) OR TS=(bacterial-infections) OR TS=(virus-diseases) OR TS=(parasitic-diseases) OR TS=(mycoses) OR TS=(central-nervous-system-infections) OR TS=(zoonoses) OR TS=(zoonosis) OR TS=(vaccine-preventable-diseases) OR TS=(sexually-transmitted-diseases) OR TS=(std) OR TS=(sexually-transmitted-agents) OR TS=(quarantinable-diseases) OR TS=(gastrointestinal-diseases) OR TS=(vector-borne-diseases) | 1,915,626 |
| #4 | TS=(plague) OR TS=(cholera) OR TS=(severe-acute-respiratory-syndrome) OR TS=(SARS) OR TS=(acquired-immune-deficiency-syndrome) OR TS=(AIDS) OR TS=(HIV) OR TS=(hepatitis) OR TS=(hepatitis-A) OR TS=(hepatitis B) OR TS=(hepatitis-C) OR TS=(hepatitis-D) OR TS=(hepatitis-E) OR TS=(unspecified-hepatitis) OR TS=(other-hepatitis) OR TS=(poliomyelitis) OR TS=(human-infections-of-highly-pathogenic-avian-influenza) OR TS=(Influenza-A) OR TS=(H1N1) OR TS=(H5N1) OR TS=(H7N9) OR TS=(measles) OR TS=(epidemic-hemorrhagic-fever) OR TS=(EHF) OR TS=(rabies) OR TS=(epidemic-encephalitis-B) OR TS=(EEB) OR TS=(Japanese-encephalitis) OR TS=(dengue) OR TS=(anthrax) OR TS=(dysentery) OR TS=(tuberculosis) OR TS=(typhoid) OR TS=(paratyphoid) OR TS=(meningitis) OR TS=(pertussis) OR TS=(diphtheria) OR TS=(neonatal-tetanus) OR TS=(scarlet-fever) OR TS=(brucellosis) OR TS=(gonorrhea) OR TS=(syphilis) OR TS=(leptospirosis) OR TS=(schistosomiasis) OR TS=(malaria) OR TS=(corona-virus disease-2019) OR TS=(COVID-19) OR TS=(influenza) OR TS=(mumps) OR TS=(rubella) OR TS=(acute-hemorrhagic-conjunctivitis) OR TS=(AHC) OR TS=(leprosy) OR TS=(typhus) OR TS=(kala-azar) OR TS=(leishmaniasis) OR TS=(echinococcosis) OR TS=(filariasis) OR TS=(infectious-diarrhea) OR TS=("Hand, foot and mouth disease") OR TS=(HFMD) | 2,052,267 |
| #5 | #3 OR #4 | 3,343,821 |
| #6 | #1 AND #2 AND #5 | 6,156 |
| #7 | #6 Filters: DOP from 2004-01-01 to 2022-01-01 | 5,836 |

**Database 2: PubMed (NCBI)**

| No. | Query | Results |
| --- | --- | --- |
| #1 | (China[Title/Abstract]) OR (Chinese[Title/Abstract]) | 409,031 |
| #2 | (((((((space[Title/Abstract]) OR (spatial[Title/Abstract])) OR (space-time[Title/Abstract])) OR (spatio-temporal[Title/Abstract])) OR (geographic[Title/Abstract])) OR (geographical[Title/Abstract])) OR (GIS[Title/Abstract])) OR (mapping[Title/Abstract]) | 947,812 |
| #3 | ((((((((((((((((((infectious diseases[Title/Abstract]) OR (communicable diseases[Title/Abstract])) OR (infection[Title/Abstract])) OR (emerging infectious diseases[Title/Abstract])) OR (epidemic[Title/Abstract])) OR (bacterial infections[Title/Abstract])) OR (virus diseases[Title/Abstract])) OR (parasitic diseases[Title/Abstract])) OR (mycoses[Title/Abstract])) OR (central nervous system infections[Title/Abstract])) OR (zoonoses[Title/Abstract])) OR (zoonosis[Title/Abstract])) OR (vaccine preventable diseases[Title/Abstract])) OR (sexually transmitted diseases[Title/Abstract])) OR (std[Title/Abstract])) OR (sexually transmitted agents[Title/Abstract])) OR (quarantinable diseases[Title/Abstract])) OR (gastrointestinal diseases[Title/Abstract])) OR (vector-borne diseases[Title/Abstract]) | 1,411,650 |
| #4 | (((((((((((((((((((((((((((((((((((((((((((((((((((((((((((plague[Title/Abstract]) OR (cholera[Title/Abstract])) OR (severe acute respiratory syndrome[Title/Abstract])) OR (SARS[Title/Abstract])) OR (acquired immune deficiency syndrome[Title/Abstract])) OR (AIDS[Title/Abstract])) OR (HIV[Title/Abstract])) OR (hepatitis[Title/Abstract])) OR (hepatitis A[Title/Abstract])) OR (hepatitis B[Title/Abstract])) OR (hepatitis C[Title/Abstract])) OR (hepatitis D[Title/Abstract])) OR (hepatitis E[Title/Abstract])) OR (unspecified hepatitis[Title/Abstract])) OR (other hepatitis[Title/Abstract])) OR (poliomyelitis[Title/Abstract])) OR (human infections of highly pathogenic avian influenza[Title/Abstract])) OR (Influenza A[Title/Abstract])) OR (H1N1[Title/Abstract])) OR (H5N1[Title/Abstract])) OR (H7N9[Title/Abstract])) OR (measles[Title/Abstract])) OR (epidemic hemorrhagic fever[Title/Abstract])) OR (EHF[Title/Abstract])) OR (rabies[Title/Abstract])) OR (epidemic encephalitis B[Title/Abstract])) OR (EEB[Title/Abstract])) OR (Japanese encephalitis[Title/Abstract])) OR (dengue[Title/Abstract])) OR (anthrax[Title/Abstract])) OR (dysentery[Title/Abstract])) OR (tuberculosis[Title/Abstract])) OR (typhoid[Title/Abstract])) OR (paratyphoid[Title/Abstract])) OR (meningitis/ pertussis[Title/Abstract])) OR (diphtheria[Title/Abstract])) OR (neonatal tetanus[Title/Abstract])) OR (scarlet fever[Title/Abstract])) OR (brucellosis[Title/Abstract])) OR (gonorrhea[Title/Abstract])) OR (syphilis[Title/Abstract])) OR (leptospirosis[Title/Abstract])) OR (schistosomiasis[Title/Abstract])) OR (malaria[Title/Abstract])) OR (corona virus disease 2019[Title/Abstract])) OR (COVID-19[Title/Abstract])) OR (influenza[Title/Abstract])) OR (mumps[Title/Abstract])) OR (rubella[Title/Abstract])) OR (acute hemorrhagic conjunctivitis[Title/Abstract])) OR (AHC[Title/Abstract])) OR (leprosy[Title/Abstract])) OR (typhus[Title/Abstract])) OR (kala azar[Title/Abstract])) OR (leishmaniasis[Title/Abstract])) OR (echinococcosis[Title/Abstract])) OR (filariasis[Title/Abstract])) OR (infectious diarrhea[Title/Abstract])) OR (Hand, foot, mouth disease[Title/Abstract])) OR (HFMD[Title/Abstract]) | 1,466,578 |
| #5 | #3 OR #4 | 2,445,157 |
| #6 | #1 AND #2 AND #5 | 3,420 |
| #7 | #6 Filters: from 2004 - 2022 | 3,123 |

**Database 3: EMBASE**

| No. | Query | Results |
| --- | --- | --- |
| #1 | (china:ti,ab,kw OR chinese:ti,ab,kw OR 'china'/exp OR 'chinese'/exp) AND [embase]/lim AND [2004-2021]/py | 394,742 |
| #2 | (space:ti,ab,kw OR spatial:ti,ab,kw OR 'space time':ti,ab,kw OR 'spatio temporal':ti,ab,kw OR geographic:ti,ab,kw OR geographical:ti,ab,kw OR gis:ti,ab,kw OR mapping:ti,ab,kw OR 'spatiotemporal analysis'/exp OR 'spatial analysis'/exp) AND [embase]/lim AND [2004-2021]/py | 612,201 |
| #3 | (epidemic:ti,ab,kw OR 'infectious diseases':ti,ab,kw OR 'communicable disease':ti,ab,kw OR infection:ti,ab,kw OR 'emerging infectious diseases':ti,ab,kw OR 'bacterial infections':ti,ab,kw OR 'virus diseases':ti,ab,kw OR 'parasitic diseases':ti,ab,kw OR mycoses:ti,ab,kw OR 'central nervous system infections':ti,ab,kw OR zoonoses:ti,ab,kw OR zoonosis:ti,ab,kw OR 'vaccine preventable diseases':ti,ab,kw OR 'sexually transmitted diseases':ti,ab,kw OR std:ti,ab,kw OR 'sexually transmitted agents':ti,ab,kw OR 'quarantinable diseases':ti,ab,kw OR 'gastrointestinal diseases':ti,ab,kw OR 'vector-borne diseases':ti,ab,kw OR 'epidemic'/exp OR 'infectious diseases'/exp OR 'communicable disease'/exp OR 'infection'/exp OR 'bacterial infections'/exp OR 'virus diseases'/exp OR 'parasitic diseases'/exp OR 'mycoses'/exp OR 'central nervous system infections'/exp OR 'zoonoses'/exp OR 'zoonosis'/exp OR 'vaccine preventable diseases'/exp OR 'sexually transmitted diseases'/exp OR 'std'/exp OR 'gastrointestinal diseases'/exp OR 'vector-borne diseases'/exp) AND [embase]/lim AND [2004-2021]/py | 2,540,983 |
| #4 | (plague:ti,ab,kw OR cholera:ti,ab,kw OR 'severe acute respiratory syndrome':ti,ab,kw OR sars:ti,ab,kw OR 'acquired immune deficiency syndrome':ti,ab,kw OR aids:ti,ab,kw OR hiv:ti,ab,kw OR hepatitis:ti,ab,kw OR 'hepatitis a':ti,ab,kw OR 'hepatitis b':ti,ab,kw OR 'hepatitis c':ti,ab,kw OR 'hepatitis d':ti,ab,kw OR 'hepatitis e':ti,ab,kw OR 'unspecified hepatitis':ti,ab,kw OR 'other hepatitis':ti,ab,kw OR poliomyelitis:ti,ab,kw OR 'human infections of highly pathogenic avian influenza':ti,ab,kw OR 'influenza a':ti,ab,kw OR h1n1:ti,ab,kw OR h5n1:ti,ab,kw OR h7n9:ti,ab,kw OR measles:ti,ab,kw OR 'epidemic hemorrhagic fever':ti,ab,kw OR ehf:ti,ab,kw OR rabies:ti,ab,kw OR 'epidemic encephalitis b':ti,ab,kw OR eeb:ti,ab,kw OR 'japanese encephalitis':ti,ab,kw OR dengue:ti,ab,kw OR anthrax:ti,ab,kw OR dysentery:ti,ab,kw OR tuberculosis:ti,ab,kw OR typhoid:ti,ab,kw OR paratyphoid:ti,ab,kw OR meningitis:ti,ab,kw OR pertussis:ti,ab,kw OR diphtheria:ti,ab,kw OR 'neonatal tetanus':ti,ab,kw OR 'scarlet fever':ti,ab,kw OR brucellosis:ti,ab,kw OR gonorrhea:ti,ab,kw OR syphilis:ti,ab,kw OR leptospirosis:ti,ab,kw OR schistosomiasis:ti,ab,kw OR malaria:ti,ab,kw OR 'corona virus disease 2019':ti,ab,kw OR 'covid 19':ti,ab,kw OR influenza:ti,ab,kw OR mumps:ti,ab,kw OR rubella:ti,ab,kw OR 'acute hemorrhagic conjunctivitis':ti,ab,kw OR ahc:ti,ab,kw OR leprosy:ti,ab,kw OR typhus:ti,ab,kw OR 'kala azar':ti,ab,kw OR leishmaniasis:ti,ab,kw OR echinococcosis:ti,ab,kw OR filariasis:ti,ab,kw OR 'infectious diarrhea':ti,ab,kw OR 'hand foot and mouth disease':ti,ab,kw OR hfmd:ti,ab,kw OR 'plague'/exp OR 'cholera'/exp OR 'severe acute respiratory syndrome'/exp OR 'sars'/exp OR 'acquired immune deficiency syndrome'/exp OR 'aids'/exp OR 'hiv'/exp OR 'hepatitis'/exp OR 'hepatitis a'/exp OR 'hepatitis b'/exp OR 'hepatitis c'/exp OR 'hepatitis d'/exp OR 'hepatitis e'/exp OR 'poliomyelitis'/exp OR 'highly pathogenic avian influenza' OR 'influenza a'/exp OR 'h1n1'/exp OR 'h5n1'/exp OR 'h7n9'/exp OR 'measles'/exp OR 'epidemic hemorrhagic fever'/exp OR 'rabies'/exp OR 'japanese encephalitis'/exp OR 'dengue'/exp OR 'anthrax'/exp OR 'dysentery'/exp OR 'tuberculosis'/exp OR 'typhoid'/exp OR 'paratyphoid'/exp OR 'meningitis'/exp OR 'pertussis'/exp OR 'diphtheria'/exp OR 'neonatal tetanus'/exp OR 'scarlet fever'/exp OR 'brucellosis'/exp OR 'gonorrhea'/exp OR 'syphilis'/exp OR 'leptospirosis'/exp OR 'schistosomiasis'/exp OR 'malaria'/exp OR 'covid 19'/exp OR 'influenza'/exp OR 'mumps'/exp OR 'rubella'/exp OR 'acute hemorrhagic conjunctivitis'/exp OR 'leprosy'/exp OR 'typhus'/exp OR 'kala azar'/exp OR 'leishmaniasis'/exp OR 'echinococcosis'/exp OR 'filariasis'/exp OR 'infectious diarrhea'/exp OR 'hand foot and mouth disease'/exp) AND [embase]/lim AND [2004-2021]/py | 1,230,951 |
| #5 | #3 OR #4 | 2,745,337 |
| #6 | #1 AND #2 AND #5 | 3,756 |

**Database 4: Cochrane Library**

| No. | Query | Results |
| --- | --- | --- |
| #1 | (China OR Chinese):ti,ab,kw | 40,491 |
| #2 | (space OR spatial OR space-time OR spatio-temporal OR geographic OR geographical OR GIS OR mapping):ti,ab,kw | 38,574 |
| #3 | (infectious diseases OR communicable diseases OR infection OR emerging infectious diseases OR epidemic OR bacterial infections OR virus diseases OR parasitic diseases OR mycoses OR central nervous system infections OR zoonoses OR zoonosis OR vaccine preventable diseases OR sexually transmitted diseases OR std OR sexually transmitted agents OR quarantinable diseases OR gastrointestinal diseases OR vector-borne diseases):ti,ab,kw | 168,853 |
| #4 | (plague OR cholera OR severe acute respiratory syndrome OR SARS OR acquired immune deficiency syndrome OR AIDS OR HIV OR hepatitis OR hepatitis A OR hepatitis B ORhepatitis C OR hepatitis D OR hepatitis E OR unspecified hepatitis OR other hepatitis OR poliomyelitis OR human infections of highly pathogenic avian influenza OR Influenza A OR H1N1 OR H5N1 OR H7N9 OR measles OR epidemic hemorrhagic fever OR EHF OR rabies OR epidemic encephalitis B OR EEB OR Japanese encephalitis OR dengue OR anthrax OR dysentery OR tuberculosis OR typhoid OR paratyphoid OR meningitis OR pertussis OR diphtheria OR neonatal tetanus OR scarlet fever OR brucellosis OR gonorrhea OR syphilis OR leptospirosis OR schistosomiasis OR malaria OR corona virus disease 2019 OR COVID-19 OR influenza OR mumps OR rubella OR acute hemorrhagic conjunctivitis OR AHC OR leprosy OR typhus OR kala azar OR leishmaniasis OR echinococcosis OR filariasis OR infectious diarrhea OR Hand, foot and mouth disease OR HFMD):ti,ab,kw | 124,736 |
| #5 | #3 OR #4 | 240,776 |
| #6 | #1 AND #2 AND #5 | 138 |
| #7 | #1 AND #2 AND #5 with Publication Year from 2004 to 2021 | 134 |

**Database 5: CAJD (CNKI)**

| No. | Query | Results |
| --- | --- | --- |
| #1 | SU='中国'+'中国的'+'我国'+'我国的' | 5,601,652 |
| #2 | SU='空间'+'时空'+'地理' | 955,555 |
| #3 | SU='传染病'+'传染性疾病'+'流行病'+'疫情' | 276,069 |
| #4 | SU='鼠疫'+'霍乱'+'新型冠状病毒肺炎'+'新冠肺炎'+'传染性非典型肺炎'+'非典'+'艾滋病'+'病毒性肝炎'+'脊髓灰质炎'+'小儿麻痹症'+'人感染高致病性禽流感'+'禽流感'+'甲流'+'H1N1'+'H5N1'+'H7N9'+'猪流感'+'麻疹'+'流行性出血热'+'出血热'+'狂犬病'+'流行性乙型脑炎'+'乙脑'+'登革热'+'炭疽'+'细菌性痢疾和阿米巴性痢疾'+'痢疾'+'肺结核'+'伤寒和副伤寒'+'伤寒'+'流行性脑脊髓膜炎'+'流脑'+'百日咳'+'白喉'+'新生儿破伤风'+'猩红热'+'布鲁氏菌病'+'布病'+'淋病'+'梅毒'+'钩端螺旋体病'+'钩体病'+'血吸虫病'+'疟疾'+'流行性感冒'+'流感'+'流行性腮腺炎'+'腮腺炎'+'风疹'+'急性出血性结膜炎'+'结膜炎'+'麻风病'+'斑疹伤寒'+'黑热病'+'包虫病'+'丝虫病'+'感染性腹泻'+'手足口病' | 507,982 |
| #5 | #3 OR #4  SU='传染病'+'传染性疾病'+'流行病'+'疫情' OR SU='鼠疫'+'霍乱'+'新型冠状病毒肺炎'+'新冠肺炎'+'传染性非典型肺炎'+'非典'+'艾滋病'+'病毒性肝炎'+'脊髓灰质炎'+'小儿麻痹症'+'人感染高致病性禽流感'+'禽流感'+'甲流'+'H1N1'+'H5N1'+'H7N9'+'猪流感'+'麻疹'+'流行性出血热'+'出血热'+'狂犬病'+'流行性乙型脑炎'+'乙脑'+'登革热'+'炭疽'+'细菌性痢疾和阿米巴性痢疾'+'痢疾'+'肺结核'+'伤寒和副伤寒'+'伤寒'+'流行性脑脊髓膜炎'+'流脑'+'百日咳'+'白喉'+'新生儿破伤风'+'猩红热'+'布鲁氏菌病'+'布病'+'淋病'+'梅毒'+'钩端螺旋体病'+'钩体病'+'血吸虫病'+'疟疾'+'流行性感冒'+'流感'+'流行性腮腺炎'+'腮腺炎'+'风疹'+'急性出血性结膜炎'+'结膜炎'+'麻风病'+'斑疹伤寒'+'黑热病'+'包虫病'+'丝虫病'+'感染性腹泻'+'手足口病' | 704,627 |
| #6 | #1 AND #2 AND #5  SU='中国'+'中国的'+'我国'+'我国的' AND SU='空间'+'时空'+'地理' AND (SU='传染病'+'传染性疾病'+'流行病'+'疫情' OR SU='鼠疫'+'霍乱'+'新型冠状病毒肺炎'+'新冠肺炎'+'传染性非典型肺炎'+'非典'+'艾滋病'+'病毒性肝炎'+'脊髓灰质炎'+'小儿麻痹症'+'人感染高致病性禽流感'+'禽流感'+'甲流'+'H1N1'+'H5N1'+'H7N9'+'猪流感'+'麻疹'+'流行性出血热'+'出血热'+'狂犬病'+'流行性乙型脑炎'+'乙脑'+'登革热'+'炭疽'+'细菌性痢疾和阿米巴性痢疾'+'痢疾'+'肺结核'+'伤寒和副伤寒'+'伤寒'+'流行性脑脊髓膜炎'+'流脑'+'百日咳'+'白喉'+'新生儿破伤风'+'猩红热'+'布鲁氏菌病'+'布病'+'淋病'+'梅毒'+'钩端螺旋体病'+'钩体病'+'血吸虫病'+'疟疾'+'流行性感冒'+'流感'+'流行性腮腺炎'+'腮腺炎'+'风疹'+'急性出血性结膜炎'+'结膜炎'+'麻风病'+'斑疹伤寒'+'黑热病'+'包虫病'+'丝虫病'+'感染性腹泻'+'手足口病') | 643 |
| #7 | #6 AND YE BETWEEN ('2004', '2021')  SU='中国'+'中国的'+'我国'+'我国的' AND SU='空间'+'时空'+'地理' AND (SU='传染病'+'传染性疾病'+'流行病'+'疫情' OR SU='鼠疫'+'霍乱'+'新型冠状病毒肺炎'+'新冠肺炎'+'传染性非典型肺炎'+'非典'+'艾滋病'+'病毒性肝炎'+'脊髓灰质炎'+'小儿麻痹症'+'人感染高致病性禽流感'+'禽流感'+'甲流'+'H1N1'+'H5N1'+'H7N9'+'猪流感'+'麻疹'+'流行性出血热'+'出血热'+'狂犬病'+'流行性乙型脑炎'+'乙脑'+'登革热'+'炭疽'+'细菌性痢疾和阿米巴性痢疾'+'痢疾'+'肺结核'+'伤寒和副伤寒'+'伤寒'+'流行性脑脊髓膜炎'+'流脑'+'百日咳'+'白喉'+'新生儿破伤风'+'猩红热'+'布鲁氏菌病'+'布病'+'淋病'+'梅毒'+'钩端螺旋体病'+'钩体病'+'血吸虫病'+'疟疾'+'流行性感冒'+'流感'+'流行性腮腺炎'+'腮腺炎'+'风疹'+'急性出血性结膜炎'+'结膜炎'+'麻风病'+'斑疹伤寒'+'黑热病'+'包虫病'+'丝虫病'+'感染性腹泻'+'手足口病') AND YE BETWEEN ('2004', '2021') | 558 |

**Database 6: CSPD (WanFang Data)**

| No. | Query | Results |
| --- | --- | --- |
| #1 | 主题:("中国" or "中国的" or "我国" or "我国的") | 14,352,280 |
| #2 | 主题:("空间" or "时空" or "地理") | 2,771,052 |
| #3 | 主题:("传染病" or "传染性疾病" or "流行病" or "疫情") | 569,103 |
| #4 | 主题:("鼠疫" or "霍乱" or "新型冠状病毒肺炎" or "新冠肺炎" or "传染性非典型肺炎" or "非典" or "艾滋病" or "病毒性肝炎" or "脊髓灰质炎" or "小儿麻痹症" or "人感染高致病性禽流感" or "禽流感" or "甲流" or "H1N1" or "H5N1" or "H7N9" or "猪流感" or "麻疹" or "流行性出血热" or "出血热" or "狂犬病" or "流行性乙型脑炎" or "乙脑" or "登革热" or "炭疽" or "细菌性痢疾和阿米巴性痢疾" or "痢疾" or "肺结核" or "伤寒和副伤寒" or "伤寒" or "流行性脑脊髓膜炎" or "流脑" or "百日咳" or "白喉" or "新生儿破伤风" or "猩红热" or "布鲁氏菌病" or "布病" or "淋病" or "梅毒" or "钩端螺旋体病" or "钩体病" or "血吸虫病" or "疟疾" or "流行性感冒" or "流感" or "流行性腮腺炎" or "腮腺炎" or "风疹" or "急性出血性结膜炎" or "结膜炎" or "麻风病" or "斑疹伤寒" or "黑热病" or "包虫病" or "丝虫病" or "感染性腹泻" or "手足口病") | 806,639 |
| #5 | #3 OR #4  主题:("传染病" or "传染性疾病" or "流行病" or "疫情") or 主题:("鼠疫" or "霍乱" or "新型冠状病毒肺炎" or "新冠肺炎" or "传染性非典型肺炎" or "非典" or "艾滋病" or "病毒性肝炎" or "脊髓灰质炎" or "小儿麻痹症" or "人感染高致病性禽流感" or "禽流感" or "甲流" or "H1N1" or "H5N1" or "H7N9" or "猪流感" or "麻疹" or "流行性出血热" or "出血热" or "狂犬病" or "流行性乙型脑炎" or "乙脑" or "登革热" or "炭疽" or "细菌性痢疾和阿米巴性痢疾" or "痢疾" or "肺结核" or "伤寒和副伤寒" or "伤寒" or "流行性脑脊髓膜炎" or "流脑" or "百日咳" or "白喉" or "新生儿破伤风" or "猩红热" or "布鲁氏菌病" or "布病" or "淋病" or "梅毒" or "钩端螺旋体病" or "钩体病" or "血吸虫病" or "疟疾" or "流行性感冒" or "流感" or "流行性腮腺炎" or "腮腺炎" or "风疹" or "急性出血性结膜炎" or "结膜炎" or "麻风病" or "斑疹伤寒" or "黑热病" or "包虫病" or "丝虫病" or "感染性腹泻" or "手足口病") | 1,181,043 |
| #6 | #1 AND #2 AND #5  主题:("中国" or "中国的" or "我国" or "我国的") and ("空间" or "时空" or "地理") and ("传染病" or "传染性疾病" or "流行病" or "疫情" or "鼠疫" or "霍乱" or "新型冠状病毒肺炎" or "新冠肺炎" or "传染性非典型肺炎" or "非典" or "艾滋病" or "病毒性肝炎" or "脊髓灰质炎" or "小儿麻痹症" or "人感染高致病性禽流感" or "禽流感" or "甲流" or "H1N1" or "H5N1" or "H7N9" or "猪流感" or "麻疹" or "流行性出血热" or "出血热" or "狂犬病" or "流行性乙型脑炎" or "乙脑" or "登革热" or "炭疽" or "细菌性痢疾和阿米巴性痢疾" or "痢疾" or "肺结核" or "伤寒和副伤寒" or "伤寒" or "流行性脑脊髓膜炎" or "流脑" or "百日咳" or "白喉" or "新生儿破伤风" or "猩红热" or "布鲁氏菌病" or "布病" or "淋病" or "梅毒" or "钩端螺旋体病" or "钩体病" or "血吸虫病" or "疟疾" or "流行性感冒" or "流感" or "流行性腮腺炎" or "腮腺炎" or "风疹" or "急性出血性结膜炎" or "结膜炎" or "麻风病" or "斑疹伤寒" or "黑热病" or "包虫病" or "丝虫病" or "感染性腹泻" or "手足口病") | 5,585 |
| #7 | #6 and Date:2004-2021  (主题:("中国" or "中国的" or "我国" or "我国的") and ("空间" or "时空" or "地理") and ("传染病" or "传染性疾病" or "流行病" or "疫情" or "鼠疫" or "霍乱" or "新型冠状病毒肺炎" or "新冠肺炎" or "传染性非典型肺炎" or "非典" or "艾滋病" or "病毒性肝炎" or "脊髓灰质炎" or "小儿麻痹症" or "人感染高致病性禽流感" or "禽流感" or "甲流" or "H1N1" or "H5N1" or "H7N9" or "猪流感" or "麻疹" or "流行性出血热" or "出血热" or "狂犬病" or "流行性乙型脑炎" or "乙脑" or "登革热" or "炭疽" or "细菌性痢疾和阿米巴性痢疾" or "痢疾" or "肺结核" or "伤寒和副伤寒" or "伤寒" or "流行性脑脊髓膜炎" or "流脑" or "百日咳" or "白喉" or "新生儿破伤风" or "猩红热" or "布鲁氏菌病" or "布病" or "淋病" or "梅毒" or "钩端螺旋体病" or "钩体病" or "血吸虫病" or "疟疾" or "流行性感冒" or "流感" or "流行性腮腺炎" or "腮腺炎" or "风疹" or "急性出血性结膜炎" or "结膜炎" or "麻风病" or "斑疹伤寒" or "黑热病" or "包虫病" or "丝虫病" or "感染性腹泻" or "手足口病")) and Date:2004-2021 | 3,045 |

**Database 7: SinoMed(CBM)**

| No. | Query | Results |
| --- | --- | --- |
| #1 | "中国"[常用字段:智能] OR "中国的"[常用字段:智能] OR "我国"[常用字段:智能] OR "我国的"[常用字段:智能] | 653,579 |
| #2 | "空间"[常用字段:智能] OR "时空"[常用字段:智能] OR "地理"[常用字段:智能] | 77,234 |
| #3 | "传染病"[常用字段:智能] OR "传染性疾病"[常用字段:智能] OR "流行病"[常用字段:智能] OR "疫情"[常用字段:智能] | 490,043 |
| #4 | "鼠疫"[常用字段:智能] OR "霍乱"[常用字段:智能] OR "新型冠状病毒肺炎"[常用字段:智能] OR "新冠肺炎"[常用字段:智能] OR "传染性非典型肺炎"[常用字段:智能] OR "非典"[常用字段:智能] OR "艾滋病"[常用字段:智能] OR "病毒性肝炎"[常用字段:智能] OR "脊髓灰质炎"[常用字段:智能] OR "小儿麻痹症"[常用字段:智能] OR "人感染高致病性禽流感"[常用字段:智能] OR "禽流感"[常用字段:智能] OR "甲流"[常用字段:智能] OR "H1N1"[常用字段:智能] OR "H5N1"[常用字段:智能] OR "H7N9"[常用字段:智能] OR "猪流感"[常用字段:智能] OR "麻疹"[常用字段:智能] OR "流行性出血热"[常用字段:智能] OR "出血热"[常用字段:智能] OR "狂犬病"[常用字段:智能] OR "流行性乙型脑炎"[常用字段:智能] OR "乙脑"[常用字段:智能] OR "登革热"[常用字段:智能] OR "炭疽"[常用字段:智能] OR "细菌性痢疾和阿米巴性痢疾"[常用字段:智能] OR "痢疾"[常用字段:智能] OR "肺结核"[常用字段:智能] OR "伤寒和副伤寒"[常用字段:智能] OR "伤寒"[常用字段:智能] OR "流行性脑脊髓膜炎"[常用字段:智能] OR "流脑"[常用字段:智能] OR "百日咳"[常用字段:智能] OR "白喉"[常用字段:智能] OR "新生儿破伤风"[常用字段:智能] OR "猩红热"[常用字段:智能] OR "布鲁氏菌病"[常用字段:智能] OR "布病"[常用字段:智能] OR "淋病"[常用字段:智能] OR "梅毒"[常用字段:智能] OR "钩端螺旋体病"[常用字段:智能] OR "钩体病"[常用字段:智能] OR "血吸虫病"[常用字段:智能] OR "疟疾"[常用字段:智能] OR "流行性感冒"[常用字段:智能] OR "流感"[常用字段:智能] OR "流行性腮腺炎"[常用字段:智能] OR "腮腺炎"[常用字段:智能] OR "风疹"[常用字段:智能] OR "急性出血性结膜炎"[常用字段:智能] OR "结膜炎"[常用字段:智能] OR "麻风病"[常用字段:智能] OR "斑疹伤寒"[常用字段:智能] OR "黑热病"[常用字段:智能] OR "包虫病"[常用字段:智能] OR "丝虫病"[常用字段:智能] OR "感染性腹泻"[常用字段:智能] OR "手足口病"[常用字段:智能] | 451,887 |
| #5 | #3 OR #4  ("鼠疫"[常用字段:智能] OR "霍乱"[常用字段:智能] OR "新型冠状病毒肺炎"[常用字段:智能] OR "新冠肺炎"[常用字段:智能] OR "传染性非典型肺炎"[常用字段:智能] OR "非典"[常用字段:智能] OR "艾滋病"[常用字段:智能] OR "病毒性肝炎"[常用字段:智能] OR "脊髓灰质炎"[常用字段:智能] OR "小儿麻痹症"[常用字段:智能] OR "人感染高致病性禽流感"[常用字段:智能] OR "禽流感"[常用字段:智能] OR "甲流"[常用字段:智能] OR "H1N1"[常用字段:智能] OR "H5N1"[常用字段:智能] OR "H7N9"[常用字段:智能] OR "猪流感"[常用字段:智能] OR "麻疹"[常用字段:智能] OR "流行性出血热"[常用字段:智能] OR "出血热"[常用字段:智能] OR "狂犬病"[常用字段:智能] OR "流行性乙型脑炎"[常用字段:智能] OR "乙脑"[常用字段:智能] OR "登革热"[常用字段:智能] OR "炭疽"[常用字段:智能] OR "细菌性痢疾和阿米巴性痢疾"[常用字段:智能] OR "痢疾"[常用字段:智能] OR "肺结核"[常用字段:智能] OR "伤寒和副伤寒"[常用字段:智能] OR "伤寒"[常用字段:智能] OR "流行性脑脊髓膜炎"[常用字段:智能] OR "流脑"[常用字段:智能] OR "百日咳"[常用字段:智能] OR "白喉"[常用字段:智能] OR "新生儿破伤风"[常用字段:智能] OR "猩红热"[常用字段:智能] OR "布鲁氏菌病"[常用字段:智能] OR "布病"[常用字段:智能] OR "淋病"[常用字段:智能] OR "梅毒"[常用字段:智能] OR "钩端螺旋体病"[常用字段:智能] OR "钩体病"[常用字段:智能] OR "血吸虫病"[常用字段:智能] OR "疟疾"[常用字段:智能] OR "流行性感冒"[常用字段:智能] OR "流感"[常用字段:智能] OR "流行性腮腺炎"[常用字段:智能] OR "腮腺炎"[常用字段:智能] OR "风疹"[常用字段:智能] OR "急性出血性结膜炎"[常用字段:智能] OR "结膜炎"[常用字段:智能] OR "麻风病"[常用字段:智能] OR "斑疹伤寒"[常用字段:智能] OR "黑热病"[常用字段:智能] OR "包虫病"[常用字段:智能] OR "丝虫病"[常用字段:智能] OR "感染性腹泻"[常用字段:智能] OR "手足口病"[常用字段:智能]) OR ("传染病"[常用字段:智能] OR "传染性疾病"[常用字段:智能] OR "流行病"[常用字段:智能] OR "疫情"[常用字段:智能]) | 811,267 |
| #6 | #1 AND #2 AND #5  (("鼠疫"[常用字段:智能] OR "霍乱"[常用字段:智能] OR "新型冠状病毒肺炎"[常用字段:智能] OR "新冠肺炎"[常用字段:智能] OR "传染性非典型肺炎"[常用字段:智能] OR "非典"[常用字段:智能] OR "艾滋病"[常用字段:智能] OR "病毒性肝炎"[常用字段:智能] OR "脊髓灰质炎"[常用字段:智能] OR "小儿麻痹症"[常用字段:智能] OR "人感染高致病性禽流感"[常用字段:智能] OR "禽流感"[常用字段:智能] OR "甲流"[常用字段:智能] OR "H1N1"[常用字段:智能] OR "H5N1"[常用字段:智能] OR "H7N9"[常用字段:智能] OR "猪流感"[常用字段:智能] OR "麻疹"[常用字段:智能] OR "流行性出血热"[常用字段:智能] OR "出血热"[常用字段:智能] OR "狂犬病"[常用字段:智能] OR "流行性乙型脑炎"[常用字段:智能] OR "乙脑"[常用字段:智能] OR "登革热"[常用字段:智能] OR "炭疽"[常用字段:智能] OR "细菌性痢疾和阿米巴性痢疾"[常用字段:智能] OR "痢疾"[常用字段:智能] OR "肺结核"[常用字段:智能] OR "伤寒和副伤寒"[常用字段:智能] OR "伤寒"[常用字段:智能] OR "流行性脑脊髓膜炎"[常用字段:智能] OR "流脑"[常用字段:智能] OR "百日咳"[常用字段:智能] OR "白喉"[常用字段:智能] OR "新生儿破伤风"[常用字段:智能] OR "猩红热"[常用字段:智能] OR "布鲁氏菌病"[常用字段:智能] OR "布病"[常用字段:智能] OR "淋病"[常用字段:智能] OR "梅毒"[常用字段:智能] OR "钩端螺旋体病"[常用字段:智能] OR "钩体病"[常用字段:智能] OR "血吸虫病"[常用字段:智能] OR "疟疾"[常用字段:智能] OR "流行性感冒"[常用字段:智能] OR "流感"[常用字段:智能] OR "流行性腮腺炎"[常用字段:智能] OR "腮腺炎"[常用字段:智能] OR "风疹"[常用字段:智能] OR "急性出血性结膜炎"[常用字段:智能] OR "结膜炎"[常用字段:智能] OR "麻风病"[常用字段:智能] OR "斑疹伤寒"[常用字段:智能] OR "黑热病"[常用字段:智能] OR "包虫病"[常用字段:智能] OR "丝虫病"[常用字段:智能] OR "感染性腹泻"[常用字段:智能] OR "手足口病"[常用字段:智能]) OR ("传染病"[常用字段:智能] OR "传染性疾病"[常用字段:智能] OR "流行病"[常用字段:智能] OR "疫情"[常用字段:智能])) AND ("空间"[常用字段:智能] OR "时空"[常用字段:智能] OR "地理"[常用字段:智能]) AND ("中国"[常用字段:智能] OR "中国的"[常用字段:智能] OR "我国"[常用字段:智能] OR "我国的"[常用字段:智能]) | 1,927 |
| #7 | #6 AND 2004-2021[日期]  (("鼠疫"[常用字段:智能] OR "霍乱"[常用字段:智能] OR "新型冠状病毒肺炎"[常用字段:智能] OR "新冠肺炎"[常用字段:智能] OR "传染性非典型肺炎"[常用字段:智能] OR "非典"[常用字段:智能] OR "艾滋病"[常用字段:智能] OR "病毒性肝炎"[常用字段:智能] OR "脊髓灰质炎"[常用字段:智能] OR "小儿麻痹症"[常用字段:智能] OR "人感染高致病性禽流感"[常用字段:智能] OR "禽流感"[常用字段:智能] OR "甲流"[常用字段:智能] OR "H1N1"[常用字段:智能] OR "H5N1"[常用字段:智能] OR "H7N9"[常用字段:智能] OR "猪流感"[常用字段:智能] OR "麻疹"[常用字段:智能] OR "流行性出血热"[常用字段:智能] OR "出血热"[常用字段:智能] OR "狂犬病"[常用字段:智能] OR "流行性乙型脑炎"[常用字段:智能] OR "乙脑"[常用字段:智能] OR "登革热"[常用字段:智能] OR "炭疽"[常用字段:智能] OR "细菌性痢疾和阿米巴性痢疾"[常用字段:智能] OR "痢疾"[常用字段:智能] OR "肺结核"[常用字段:智能] OR "伤寒和副伤寒"[常用字段:智能] OR "伤寒"[常用字段:智能] OR "流行性脑脊髓膜炎"[常用字段:智能] OR "流脑"[常用字段:智能] OR "百日咳"[常用字段:智能] OR "白喉"[常用字段:智能] OR "新生儿破伤风"[常用字段:智能] OR "猩红热"[常用字段:智能] OR "布鲁氏菌病"[常用字段:智能] OR "布病"[常用字段:智能] OR "淋病"[常用字段:智能] OR "梅毒"[常用字段:智能] OR "钩端螺旋体病"[常用字段:智能] OR "钩体病"[常用字段:智能] OR "血吸虫病"[常用字段:智能] OR "疟疾"[常用字段:智能] OR "流行性感冒"[常用字段:智能] OR "流感"[常用字段:智能] OR "流行性腮腺炎"[常用字段:智能] OR "腮腺炎"[常用字段:智能] OR "风疹"[常用字段:智能] OR "急性出血性结膜炎"[常用字段:智能] OR "结膜炎"[常用字段:智能] OR "麻风病"[常用字段:智能] OR "斑疹伤寒"[常用字段:智能] OR "黑热病"[常用字段:智能] OR "包虫病"[常用字段:智能] OR "丝虫病"[常用字段:智能] OR "感染性腹泻"[常用字段:智能] OR "手足口病"[常用字段:智能]) OR ("传染病"[常用字段:智能] OR "传染性疾病"[常用字段:智能] OR "流行病"[常用字段:智能] OR "疫情"[常用字段:智能])) AND ("空间"[常用字段:智能] OR "时空"[常用字段:智能] OR "地理"[常用字段:智能]) AND ("中国"[常用字段:智能] OR "中国的"[常用字段:智能] OR "我国"[常用字段:智能] OR "我国的"[常用字段:智能]) AND 2004-2021[日期] | 1,743 |
